# A Multidisciplinary Approach Providing New Insight into Fruit Flesh Browning Physiology in Apple (*Malus x domestica* Borkh.)

**DOI:** 10.1371/journal.pone.0078004

**Published:** 2013-10-18

**Authors:** Mario Di Guardo, Alice Tadiello, Brian Farneti, Giorgia Lorenz, Domenico Masuero, Urska Vrhovsek, Guglielmo Costa, Riccardo Velasco, Fabrizio Costa

**Affiliations:** 1 Genomics and Crop Biology Department Research and Innovation Centre, Fondazione Edmund, Mach, San Michele all’Adige (Trento), Italy; 2 Department of Fruit Trees & Woody Plant Science, University of Bologna, Bologna, Italy; CSIRO, Australia

## Abstract

In terms of the quality of minimally processed fruit, flesh browning is fundamentally important in the development of an aesthetically unpleasant appearance, with consequent off-flavours. The development of browning depends on the enzymatic action of the polyphenol oxidase (PPO). In the ‘Golden Delicious’ apple genome ten *PPO* genes were initially identified and located on three main chromosomes (2, 5 and 10). Of these genes, one element in particular, here called *Md-PPO*, located on chromosome 10, was further investigated and genetically mapped in two apple progenies (‘Fuji x Pink Lady’ and ‘Golden Delicious x Braeburn’). Both linkage maps, made up of 481 and 608 markers respectively, were then employed to find QTL regions associated with fruit flesh browning, allowing the detection of 25 QTLs related to several browning parameters. These were distributed over six linkage groups with LOD values spanning from 3.08 to 4.99 and showed a rate of phenotypic variance from 26.1 to 38.6%. Anchoring of these intervals to the apple genome led to the identification of several genes involved in polyphenol synthesis and cell wall metabolism. Finally, the expression profile of two specific candidate genes, up and downstream of the polyphenolic pathway, namely phenylalanine ammonia lyase (PAL) and polyphenol oxidase (PPO), provided insight into flesh browning physiology. *Md-PPO* was further analyzed and two haplotypes were characterised and associated with fruit flesh browning in apple.

## Introduction

Fruit quality features, represented by the biochemical and physical properties making a fruit edible and appreciated by consumers, are nowadays considered a major priority in several breeding programmes worldwide. For this reason, in the last decade the scientific community has initiated a series of extensive studies addressed at examining the genetic and molecular mechanisms responsible for controlling the fundamental physiological processes ultimately leading to fruit quality [1-5]. In the modern system of fruit distribution and marketing, fruit quality also needs to be guaranteed during storage, allowing high quality standards to be maintained. In addition to this, pre-prepared fresh cut fruit is rapidly becoming interesting, given increased consumer demand for fresh and convenient food [6,7]. However, the fruit processing procedure can encounter serious problems, which need to be prevented in order to ensure high quality and at the same time avoid substantial loss of fruit. One of the most important problems occurring during fruit processing is flesh browning, which is undesirable due to the aesthetically unpleasant appearance and consequential off-flavour [8,9]. 

**Figure 1:**
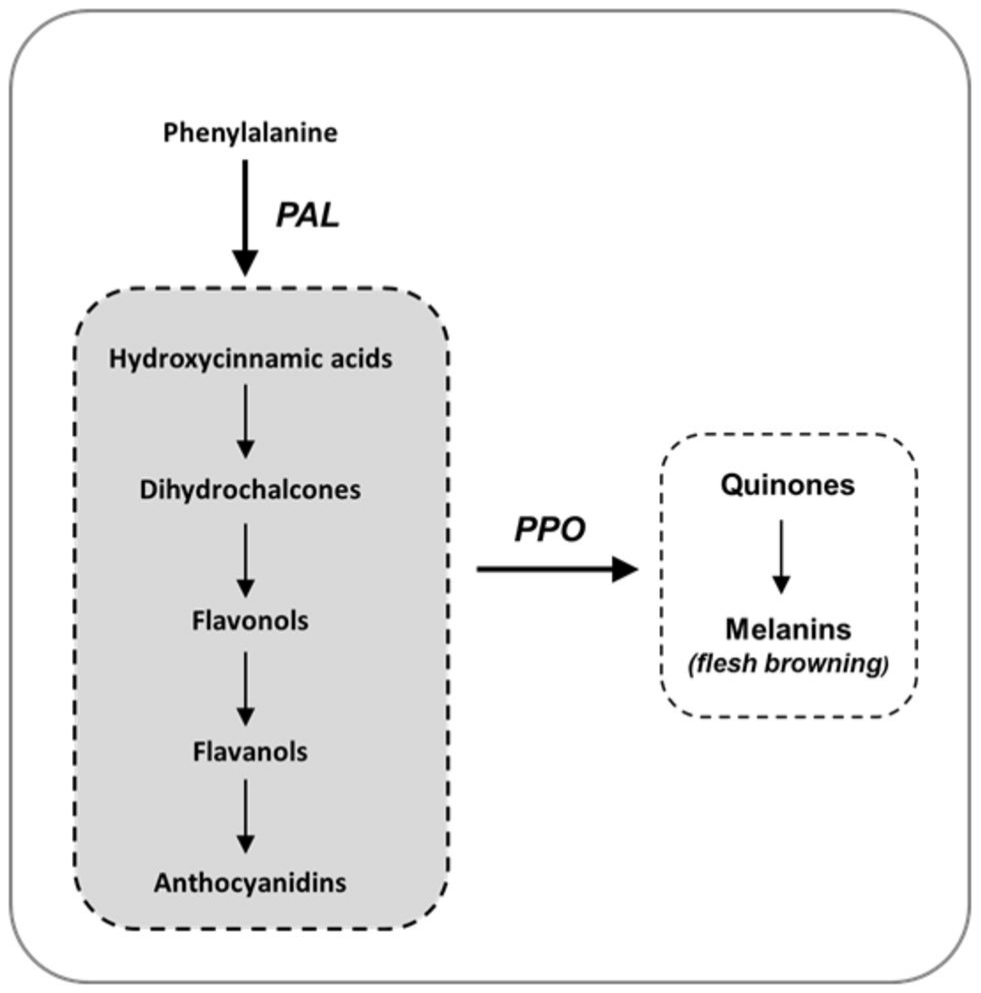
Diagram of the biosynthetic pathway for polyphenol compounds, highlighting the cascade of compounds produced from phenylalanine and the action of the PPO enzyme.

The apple’s susceptibility to flesh browning is thought to be the result of complex interplay between the polyphenoloxidase (PPO) enzyme and polyphenol content [10]. PPO is a bi-copper metalloenzyme showing two conserved copper-binding domains, CuA and CuB, which interact with molecular oxygen and phenolic substrates [11]. The PPO enzyme catalyses the formation of quinones from phenols through the hydroxylation of monophenols to o-diphenols (such as chlorogenic acid and (-)-epicatechin) and their subsequent dehydrogenation to o-quinones [12]. *O-*quinones are highly reactive, as they can undergo self-polymerisation or react with amines and thiol groups to form brown pigments (Figure 1). In the cell, after a PPO is encoded in the nucleus it is transported to the cytoplasm, where proPPO formed is stored in the plastid. The enzyme is thus physically separated from its substrate, which is located in the vacuole [12-14]. The loss of subcellular structural compartmentation, following damage or cutting, leads the PPO enzyme to come into contact with phenolic compounds. The quinones produced by oxidation rapidly condense to generate relatively insoluble brown polymers (melanins), ultimately detected as the browning reaction. For the conversion of *o-*dihydroxyphenols into *o-*benzoquinones, PPO can also use O_2_ as a second substrate (catecholase activity; [13]). 

Initial browning can also occur during storage in a controlled atmosphere, causing serious fruit loss. The importance of browning prevention depends on the fact that the symptoms in whole fruit are not usually externally visible, and thus the problem can only be detected after purchase, influencing consumer confidence. Of several strategies adopted to prevent fruit flesh browning, the application of low O_2,_ together with a high concentration of CO_2_ are the most common, although an excessive level of CO_2_ may also affect fruit quality negatively [15]. For quality control in minimally processed fruit, another system for reducing flesh browning is control of PPO activity. This enzyme can indeed be inactivated by ascorbic acid or its derivates, which reduce phenoxyl radicals and quinone forms of phenolics back to precursor phenols, through a coupled oxidation/reduction reaction [16,17]. However, the effect of ascorbic acid is temporary, as it is further irreversibly oxidized into dehydroascorbic acid, enabling browning to occur again [18]. 

Due to this mechanism, development of functional markers for the selection of new apple accessions characterised by a minimum browning phenotype would be a valuable approach, also taking into account the fact that several chemicals (such as sulphate) and physical prevention strategies are no longer accepted by the FDA agency [15,19]. To date, genetic control of flesh browning has only been partially assessed using the QTL mapping approach, revealing the presence of two intervals located on linkage groups 3 and 17 [20,21]. However, these two QTLs have not yet been shown to be associated with any candidate genes involved in the flesh browning metabolism. In the apple, the only *PPO* gene positioned to date on a genetic map is represented by [22], but its genomic location on chromosome 5 has not yet been associated with any browning phenotype.

In this survey, two independent apple populations were employed to examine and characterise fruit flesh browning in apple. A new gene, here called *Md-PPO*, was identified and associated with the different browning rate. To better understand this physiological mechanism, both transcript and metabolite assessment was carried out on a set of apple samples developing browning. Finally, a new hypothesis regarding the physiological regulation of flesh browning is discussed here.

## Materials and Methods

### Plant materials

To investigate fruit flesh browning in apple and detect QTL regions involved in control of this phenotype, two independent full-sib populations were employed. The first progeny came from the controlled cross between ‘Fuji’ and ‘Pink Lady’ (POP_1), while the second (POP_2) was created by crossing the apple cv. ‘Golden Delicious’ with ‘Braeburn’. Both populations were planted in the same experimental orchard in 2003 and both belong to the breeding programme currently underway at FEM (Foundation Edmund Mach), located in northern Italy. All the plant material was grafted onto M9 rootstock and maintained by making use of standard technical management for pruning and disease control.

### Fruit Flesh Browning Phenotyping

Fruit flesh browning in apple was assessed in the two populations at two specific stages in the fruit ripening process. Apples from POP_1 were assessed at harvest, while for POP_2 flesh browning was also measured after two months’ cold storage, to estimate the impact of low temperature in the control of this disorder. For convenience in terms of the genotyping procedure, both progenies were represented by the two parental cultivars, plus 94 seedlings each (to set a 96 well plate). The browning phenotype was assessed by measuring the flesh colour from two halves of a cut apple with a Konica Minolta digital colorimeter CM2600D. Five whole apples/genotype were considered, collecting ten measurements/samples at the end. The use of this instrument allowed digital characterisation of the flesh colour, based on the tristimulus coordinates L^*^, a^*^ and b^*^. This colour space was chosen with regards to the most popular RGB (red, green and blue) system because it provides a more uniform colour difference in relation to human perception. For this reason the L^*^ a^*^ b^*^ is the colour space generally employed in photoelectric measurements. The L^*^ value spans from 0 to 100, corresponding to black (0) and white (100), while a^*^ and b^*^ indexes do not have specific numerical limits. Negative numbers for a^*^ indicate a green shade while positive values point to magenta. For b^*^, negative and positive values are for blue and yellow respectively. Analysis of flesh browning was performed by measuring the flesh colour at three specific moments: after cutting (T_0_), after 30 minutes (T_30_) and after 60 minutes (T_60_) on the same sample. Furthermore, the absolute value for L^*^, a^*^ and b^*^ taken at these three stages (T_0_, T_30_ and T_60_) and the delta values (∆) between T_30_ and T_0_, T_60_ and T_0_ and T_60_ and T_30_ were considered. The percentage variation, represented by ((T_i_ - T_0_)/T_0_)x100, where T_i_ is the L^*^, a^*^ or b^*^ value after 30 or 60 minutes and T_0_ is the corresponding index measured after cutting, was also calculated. Finally, apple flesh browning was examined using twenty-four new colour parameters.

### PPO identification

To target the *PPO* gene set located over the apple genome assembly, BLAST searching was performed, using the only available and functionally well-characterised *PPO* gene for apple as the query, coded in the GeneBank (www.ncbi.nlm.nih.gov) as L29450 [12]. BLAST was performed using the internal resources of FEM (http://genomics.research.iasma.it) and the predicted gene set produced during the apple genome sequencing project [23]. The sequences retrieved were confirmed through gene annotation, performed using the Uniprot database (http://uniprot.org), and further compared with the one released by GDR (http://rosaceae.org). Gene distribution over the chromosomes was illustrated using MapChart [24].

### Marker development

Two types of functional molecular markers related to the *PPO* elements identified here were newly designed: SSRs and SNPs. Initially a set of SSR motifs were discovered *in silico* in the genomic contigs of each *PPO*, using Sputnik software (http://espressosoftware.com/sputnik/index.html). Primer pairs (Table S1) flanking the microsatellite motif, were designed by Primer3 software (http://frodo.wi.mit.edu/primer3/) and further used for specific amplification. SSR markers were initially exploited on the four parental cultivars to search for polymorphism in a PCR mix with a final volume of 20 μL. The PCR conditions were as follow: 5 ng of DNA, 10 X Buffer, 0.25mM dNTPs, 0.075 µM of forward labelled and reverse primers and 1000 U of 5Prime^®^ Taq polymerase. Temperature conditions were: initial denaturation at 94 °C for 150 s followed by 32 cycles at 94°C for 30 s, 58°C for 45 s, 72°C for 60 s, and a final extension at 72 °C for 5 min. Fragment analysis was performed by an ABI PRISM^®^ 3730 capillary sequencer (Applied Biosystems by Life Technologies, Carlsbad, CA, USA) in a final volume of 0.3 µL of PCR product, 9.67 µL formamide and 0.03 µL of 500-LIZ denaturated for 3 min at 95 °C. Fragment sizing was carried out with PeakScanner^®^ software (Applied Biosystems by Life Technologies, Carlsbad, CA, USA). 

 In addition to the SSR markers, a set of SNPs related to the *PPO* gene corresponding to L29450 was also created. Three pairs of primers were designed, in order to cover the full length of the entire gene: PPO_1_for:_ CTTCTTGGTCTTGGAGGTCT and PPO_1_rev:_ ATCGGAGCTTGTCGTAGAGA; PPO_2_for:_ CCACAACTCATGGCTCTTCT and PPO_2_rev:_ CTAACTCTGCTGTCTCGTTG; PPO_3_for:_ GTTCTTTGGGAACCCGTACA and PPO_3_rev:_ CATCAAACTTCACAGCCACG. Amplification of the three fragments was carried out in a final volume of 10 µL containing 5 ng of DNA, 10X buffer, 0.25 mM dNTPs, 1 µM forward and reverse primers and 1000U 5Prime^®^ Taq polymerase. PCR was carried out using an ABI 2720 Thermal Cycler (Applied Biosystems by Life Technologies, Carlsbad, CA, USA) with the following thermal conditions: denaturation at 94 °C for 2 minutes, followed by 35 cycles of denaturation at 94 °C for 45 s, annealing at 65 °C for 45 s, extension at 72 °C for 2 min, finishing with a conclusive extension at 72 °C for 10 min. Amplicons were than purified by adding 4 µl of water and 1µl of ExoSAP to 1 µL of PCR product. The mix was then incubated for 45 minutes at 37 °C, followed by 15 minutes at 75 °C. In a second step, 2 µL of Buffer 5X, 1 µL of the forward primer (at 3.2 uM), 1 µL of Big dye terminator (Applied Biosystem by Life Technologies, Carlsbad, CA, USA) and 0.5 µL of water were added to the purification mix to reach a final volume of 10 µL. The sequencing reaction was performed for 2 minutes at 96 °C followed by 39 cycles of denaturation (96 °C for 10 s), annealing (50 °C for 5 s) and extension (60 °C for 4 minutes). The sequencing runs were carried out using an ABI PRISM^®^ 3730 capillary sequencer (Applied Biosystems by Life Technologies, Carlsbad, CA, USA) and finally the SNPs were genotyped by re-sequencing the amplicons across the two populations. SNP scoring was carried out by analysing the sequences obtained using Pregap4 software (Staden package; http://staden.sourceforge.net).

### QTL analysis

The new markers designed in this study were used to improve the current version of the two molecular maps. The POP_1 map was originally created for comprehensive examination of fruit texture in apple [25], while POP_2 was employed to anchor the contigs produced during the apple genome sequencing projects [23]. Marker integration in the context of these two maps was done using JoinMap 4.0 software [26], employing the Kosambi mapping function, while visual display of the linkage groups was carried out using MapChart software [24]. Finally, genetic and phenotypic data of 47 seedlings of the POP_1 were used for QTL analysis, in order to target the genomic regions associated with apple flesh browning. QTL intervals were detected using MapQTL^®^6 [27], and the Interval Mapping (IM) algorithm. To reduce residual variance and the effect of possible false positives, the markers with the highest LOD value were selected as cofactors in the subsequent MQM computation. The threshold for calling positive QTLs was established at a LOD value of 3, after running 1000 permutations.

To examine the genetic regulation of this phenotype, each QTL interval was anchored on the assembled apple genome and the sequences of the predicted gene were retrieved by using the Computational Web Resources of FEM (www.genomics.research.iasma.it). From the geneset underlying the QTL regions, the predicted aminoacid sequences were employed to perform gene annotation by interrogating the UniProt (Viridiplantae) database.

Finally, the effect of the Md-PPO haplotype was validated on two set of seedlings belonging to the POP_2, 64 measured at harvest and 58 after two months of cold storage.

### RNA isolation and qPCR analysis

For transcript profiling, flesh samples from four replicates of the ‘Golden Delicious’ collected at T_0_ (immediately after cutting), T_30_ and T_60_ were used. Cortex tissues were initially frozen in liquid nitrogen and further ground to a fine powder. RNA extraction was performed using the Spectrum Plant total RNA kit (Sigma). RNA was quantified using a NanoDrop ND-8000 spectrophotometer (Thermo Scientific, USA), while its purity and integrity was assessed by an Agilent 2100 Bioanalyzer. The RNA isolated from the apple samples (T_0_, T_30_ and T_60_) was converted into cDNA by the ‘SuperScript VILO cDNA Synthesis Kit’ (Invitrogen). Prior to this, 1 µg of total RNA from each sample was pretreated with 2 Units of rDNAse I (DNA free kit, Ambion) and used as a starting template. To clarify the physiological regulation of flesh browning, the expression of two genes, *Md-PAL* and *Md-PPO*, was assessed by using the following specific primer pairs: *Md-PAL*_*for*_: AGACCCTCAATGCCTCAGAA *; Md-PAL*_*rev*_: CAAGCCAGAACCAACAGCAG *; Md-PPO*_*for*_: CCTACTCACAAAGCCCAAGC and *Md-PPO*_*rev*_: CCTCCAAGACCAAGAAGCAC.

Real Time PCR was performed using ACTIN as housekeeping gene (*Md-ACT*_*for*_: TGACCGAATGAGCAAGGAAATTACT; *Md-ACT*_*rev*_ *:* TACTCAGCTTTGGCAATCCACATC).

Transcript quantification, carried out using the ViiA7 instrument (Applied Biosystem), was performed by using the ‘KAPA SYBR FAST Universal qPCR kit’ (Kapa Biosystems). PCR thermal conditions were: incubation at 95°C for 20 sec, 40 cycles of 95°C 1 sec and 60°C 20 sec. Finally, a cycle at 95°C for 15 sec, 60°C for 1 min and 95°C for 15 sec was applied to determine the melting curve. The Ct results were obtained by averaging three independent normalised expression values for each sample, carried out using Q-gene software [28]. Relative gene expression was plotted as the mean of the normalised expression values of the triplicates. 

### Phenolic profiling

Phenols were extracted from the ground cortex tissues of ‘Golden Delicious’ collected at T_0_, T_30_ and T_60_, following the procedure reported in [29] and analysed as described in [30]. Briefly, 2 g of tissue powder collected from four replicates each experimental time, previously prepared for RNA isolation, were treated in sealed glass vials using 4 ml of water/methanol/chloroform solution (20:40:40). After vortexing for 1 min, the samples were mixed using an orbital shaker for 15 min at room temperature, and further centrifuged at 1000g (4 °C) for 10 min, after which the upper phases, made up of aqueous methanol extract, were collected. Extraction was repeated by adding another 2.4 ml of water/methanol (1:2) to the pellet and chloroform fractions. After the final centrifugation, the upper phases from the two extractions were combined and brought to the volume of 10 ml and filtered with a 0.2 μm PTFE filter prior to liquid chromatography-mass spectrometry analysis. Ultraperformance liquid chromatography was performed employing a Waters Acquity UPLC system (Milford, MA, USA) coupled to a Waters Xevo TQMS (Milford, MA, USA) working in ESI ionisation mode. Separation of the phenolic compounds was achieved on a Waters Acquity HSS T3 column 1.8 μm, 100 mm × 2.1 mm (Milford, MA, USA), kept at 40 °C, and with two solvents: A (water containing 0.1% formic acid) and B (acetonitrile containing 0.1% formic acid). The samples were eluted according to a linear gradient method described in detail by Vrhovsek et al. [30]. 2 μL of the final extraction were injected by an autosampler set at the temperature of 6 °C. Data were processed by using Waters MassLynx 4.1 and TargetLynx software. The compounds analysed belong to four chemical classes: hydroxycinnamic acids, dihydrochalcones, flavan-3-ols and flavonols. 

## Results and Discussion

### Distribution of apple flesh browning traits

**Figure 2:**
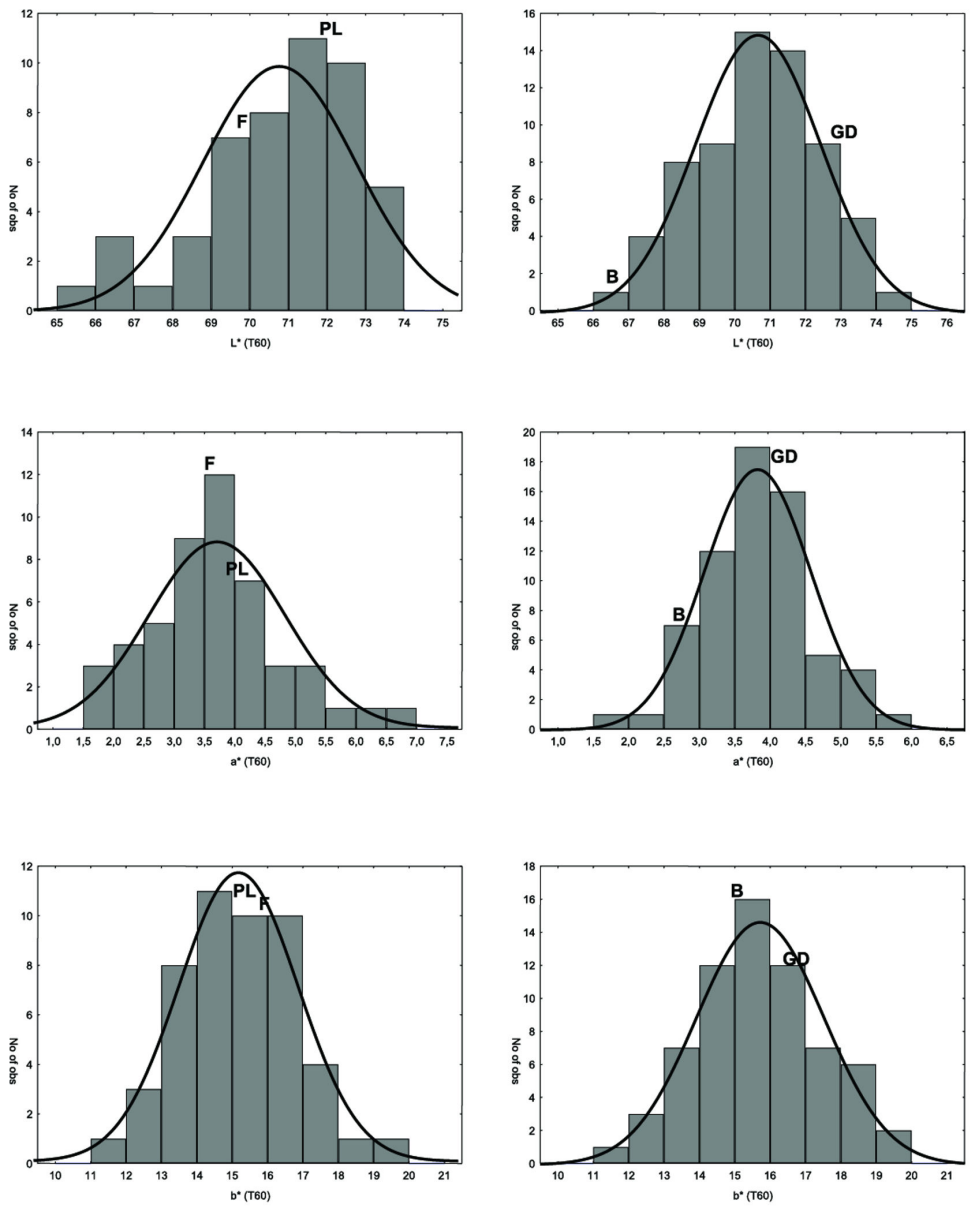
Trait distribution for L^*^, a^*^ and b^*^ parameters measured after 60 minutes of exposure to air. The panels on the left are for POP_1 (‘Fuji x Pink Lady’), while the panels on the right are for POP_2 (‘Golden Delicious x Braeburn’). The grey bars in each panel represent the data distribution observed, while the black line is the normal fit of data. The x-axis gives the value for the three indexes, while the y-axis plots the number of observations. The position of the four parental varieties is indicated by four letters: F_’Fuji’, PL_’Pink Lady’, GD_’Golden Delicious’ and B_’Braeburn’.

Flesh browning in apple is understood as the colour change in the fruit cortex after cutting. Analysis was performed immediately after cutting and after 30 and 60 minutes. Within 1 hour a significant variation in colour intensity was observed, as shown by the four parental cultivars (Figure S1 i-iv). Flesh browning was assessed in two populations (POP_1 and POP_2), and the distribution of the several parameters considered to examine the phenotype is illustrated in Figure 2. This shows the data distribution of the L^*^, a^*^ and b^*^ index absolute values, measured after 60 minutes of exposure to the air, in order to evaluate the maximum trait variability existing within each progeny. In both cases, all the browning sub-phenotypes showed quantitative distribution, which is the main prerequisite for a QTL mapping survey. However, some exceptions with skewed segregation were detected, such as L^*^ at T60. It is also worth noting that analysis of data distribution revealed a transgressive type of segregation (particularly in the POP_1). A set of seedlings exceeding the phenotypic value of the two parents was indeed observed, making the efforts to identify molecular markers suitable for the anticipated selection of this trait worthwhile. This type of segregation is also reflected in the difference of the L^*^, a^*^ and b^*^ values measured over the three stages for the four parental cultivars (Figure S1_v).

### PPO Organisation in the Apple Genome

**Figure 3:**
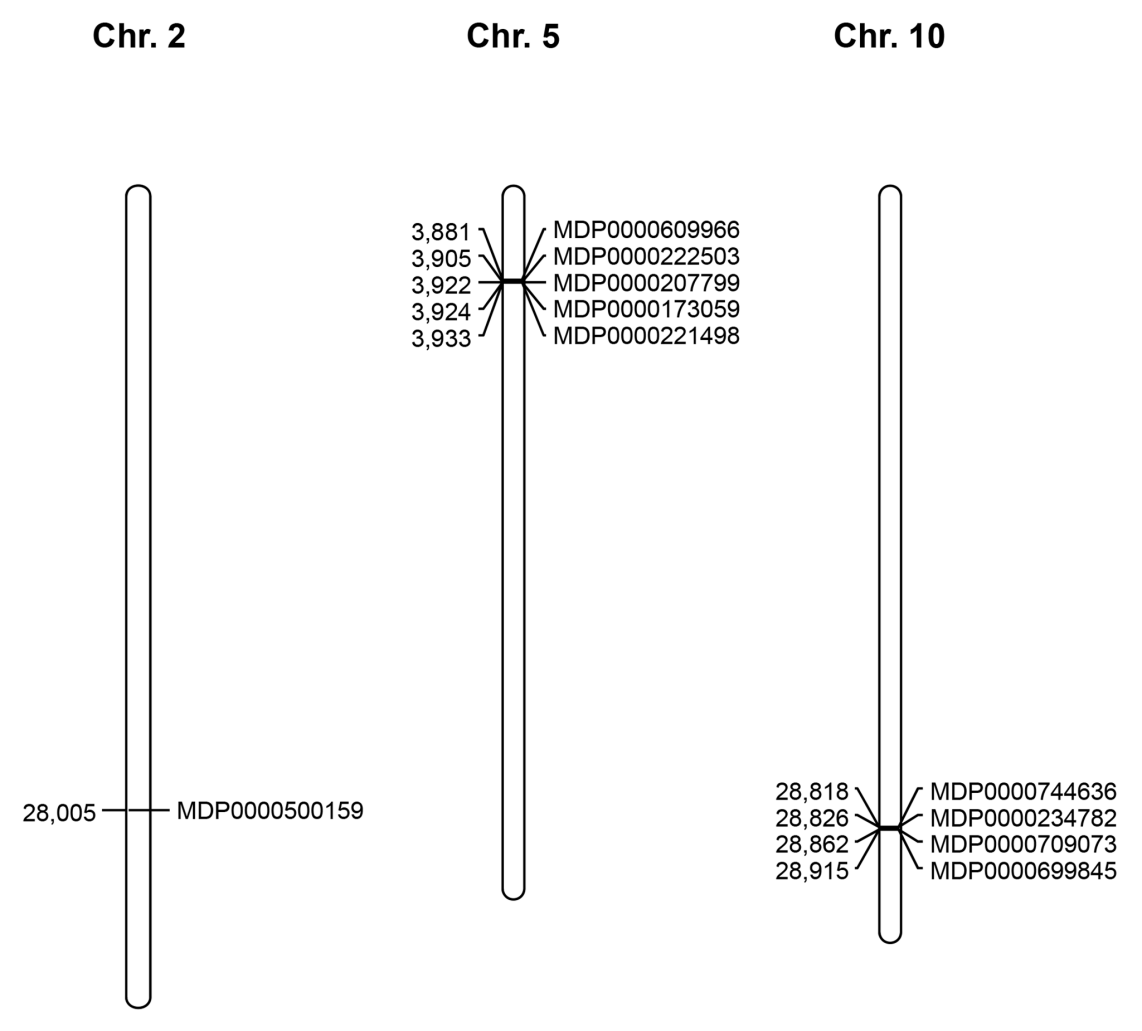
Physical localization of the ten *PPO* genes discovered in the ‘Golden Delicious’ genome. The position in Kb is plotted for each chromosome on the left , while the gene ID is shown on the right.

To identify the several *PPO* genes positioned within the apple genome assembly, the L29450 sequence was used as query, as to date it is the only *PPO* gene whose regulation has been experimentally validated for apple [12]. The predicted apple gene set was investigated using iterative nucleotide BLAST searching, and only genes showing an e-value of ≤ 2e^-04^ were further considered as possible polyphenol oxidase candidates. The resulting gene list was further annotated using BLASTp (adopting the amino acid sequences resulting from the predicted gene set) through interrogation of the Uniprot database. Of the initial set, ten genes were finally annotated as *PPOs* and located in three distinct chromosomes (Figure 3). In particular, one element was located at the bottom of chromosome 2 (MDP0000500159), five were clustered at the top of chromosome 5 (MDP0000609966, MDP0000222503, MDP0000207799, MDP0000173059 and MDP0000221498) while the last group, made up of four elements, was detected at the bottom of chromosome 10 (MDP0000744636, MDP0000234782, MDP0000709073 and MDP0000699845; available at GDR: http://www.rosaceae.org/gb/gbrowse/malus_x_domestica/ and FEM: http://genomics.research.iasma.it/gb2/gbrowse/apple). The number of *PPOs* identified for apple is also consistent with what has been reported so far for other species. In tomato, for instance, seven genes belonging to this family were positioned on chromosome 8 [31]. In this species, as observed for apple, the *PPO* elements are organised in clusters. With regards to tomato, apple shows three chromosomes characterized by the presence of *PPO* elements, but chromosome 2 contains only one element, and chromosomes 5 and 10 are homoeologous, due to the recent genome duplication occurring in this species [23]. Moreover, other authors have already presented the duplication of these two chromosomes in apple in previous publications [32-35]. Recently, a comprehensive survey performed on 25 sequenced genomes revealed that other species shared a similar number of *PPO* genes [36], such as *Sorghum bicolour* (8), *Glycine max* (11) and *Populus trichocarpa* (11). Beside this, it was also discovered that the *PPO* family can vary greatly in size across species. Indeed, *Solanum tuberosum* shows only 5 *PPO* elements [37], *Zea mays* 6 and *Vitis vinifera* 4. This difference was thought to be a consequence of duplication events, responsible, for instance, for the complete loss of this gene family in *Arabidopsis thaliana* [36,38]. The organisation of the cluster of *PPO* genes in apple is also supported by phylogenetic analysis carried out by Tran and colleagues, which suggests the tandem arrangement of *PPO* genes on chromosomes as a consequence of these recent genome duplication events. Of the total number of *PPO* genes found in the apple genome, the one encoded as MDP0000699845 was shown to be the most similar to L29450, initially used as the query, and for this reason it was further studied in more detail, to exploit its association with the fruit flesh browning rate.

### Functional marker design and QTL mapping

The first set of functional markers designed was related to microsatellite motifs discovered on the contigs where the ten *PPO* genes were targeted. Initially, these SSR markers were tested for polymorphism, searching in the four parental cultivars (‘Fuji’, ‘Pink Lady’, ‘Golden Delicious’ and ‘Braeburn’). Of these markers, only two primer pairs provided interesting results: Md-PPO_SSR_ch5e and Md-PPO_SSR_ch10d (Figures S2 and S3). The position of these two markers, assigned in silico to chromosomes 5 and 10 (as reported by the Genome Browser), was further confirmed by genetic mapping of the two populations considered in this investigation (Figure S4 and S5). Md-PPO_SSR_ch05e was mapped at the top of LG (linkage group) 5 in both progenies (at 17.4 cM in POP_1 and 4.8 cM in POP_2). In the same way, Md-PPO_SSR_ch10d was also mapped at 64.4 cM and 80 cM from the top of linkage group 10 for POP_1 and POP_2 respectively. The different genetic positions observed could be attributed to different genome coverage of these two linkage maps, being higher in POP_2.

Because SNPs found within gene sequences are considered to be one of the most frequent and important causal events controlling phenotype variation [39-41], a set of SNP markers was also exploited. It is indeed known that substitution of a single base can modify the aminoacid sequence, leading to functional variation [42]. Furthermore, given that they are the most abundant type of molecular marker within the genome, SNP markers are currently recognised as those most valuable in finding associations with phenotypic traits [43]. As proof of this, several references have already reported the use of SNPs in trait association analysis [41,44-51]. SNP discovery was performed by selecting the gene MDP0000699845 as the main candidate, on which three primer pairs were designed to characterise the full-length sequence of the gene. On aligning the sequences read in the four parental cultivars, two SNPs were exploitable in POP_2 only, being homozygous in the parents of POP_1. The two SNPs, located respectively at 170 bp and 500 bp, were genotyped by sequencing all the seedlings in the ‘Golden Delicious x Braeburn’ population, showing 1:1 segregation (χ^2^ = 0.4). Both SNPs were heterozygous in the apple cv. ‘Braeburn’ (SNP_170_: CG and SNP_500_: AG), while in ‘Golden Delicious’ the allelotype configuration was always homozygous (GG) for both SNPs. The close proximity between these two SNPs generated identical segregation (high LD level), which enabled construction of a haplotype, further positioned on the POP_2 genetic map (Figure S4). As expected the *Md-PPO* gene was mapped by means of this haplotype on LG 10 at 1.7 cM from Md-PPO_SSR_ch10d. This distance may however be overestimated, due to the fact that the SNP set used to saturate the maps came from the sequencing of the ‘Golden Delicious’ genome [52]. Because of this, the SNPs were fully informative only for the ‘Golden Delicious’ parent (“ab x aa” and “ab x ab”), while in ‘Braeburn’ the segregation type “aa x ab” was completely absent, leading to reduced representation of recombination events for this paternal cultivar in the POP_2 map. Between the two SNPs identified by comparing ‘Golden Delicious’ and ‘Braeburn’, SNP_170_ caused an amino acid substitution in the protein sequence (from Glycine in ‘Golden Delicious’ to Glutamic Acid in ‘Braeburn’; Figure S6) that may theoretically contribute towards modifying the occurrence of flesh browning observed in this progeny.

**Figure 4:**
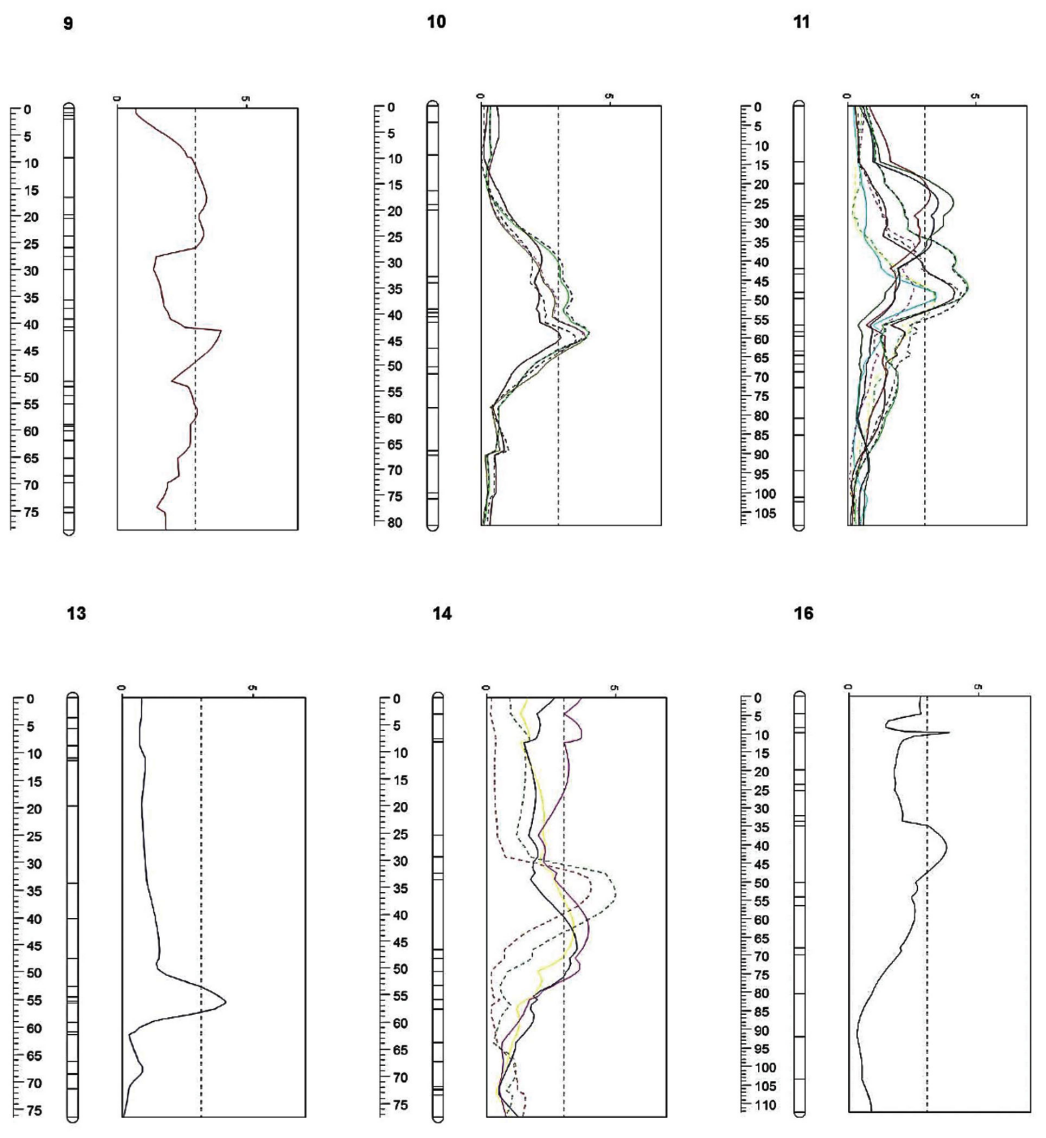
QTL-LOD profile targeted on 6 linkage groups of the POP_1 and related to several flesh browning components. In particular:. LG9: b^*^_T_0_ (solid red). LG10: L^*^T_30-0_ (solid green), a^*^_T_30-0_ (solid violet), L^*^_T_60-0_ (solid dark green), a^*^_T_60-0_ (dashed black), L^*^T_30-0_% (dashed blue), L^*^_T_60-0_% (dashed pink). LG11: a^*^_T_30_ (solid dark green), b^*^_T_30_ (solid red), a^*^_T_60_ (solid blue), L^*^_T_30-0_ (solid green), a^*^_T_30-0_ (solid violet), b^*^_T_30-0_ (solid light blue), a^*^_T_60-0_ (dashed black), L^*^_T_30-0_% (dashed blue), a^*^_T_30-0_% (dashed yellow), L^*^_T_60-0_% (dashed pink), a^*^_T_60-0_% (dashed light green). LG13: b^*^_T_30_ (solid blue). LG14: b^*^_T_30_ (solid black), a^*^_T_60_ (solid yellow), b^*^_T_60_ (solid pink), L^*^_T_60-30_ (dashed red), a^*^_T_60-30_ (dashed green). LG16: a^*^_T_0_ (solid black). The QTL analysis was performed by using the phenotypic data of 47 seedlings of the POP_1. For each seedling a total of five apples were considered (biological replicates), and for each fruit the browning was measured on the two cut halves (technical replicates).

Integration of these markers led to an improved version of the two maps, which were subsequently used for a QTL mapping survey carried out on POP_1 (composed by 481 markers for a total length of 1430.8 cM with an average distance between markers of 2.9 cM) and a further haplotype validation step performed on POP_2 (composed by 608 markers for a total length of 1204 cM with an average distance between markers of 1.9 cM). Within the POP_1 genome, six linkage groups out of seventeen (apple chromosome numbers), showed significant intervals for the presence of twenty-five QTLs associated with fruit flesh browning. Five of these were identified when a^*^ and b^*^ absolute values were measured at T_0_, T_30_ and T_60_. In this case it is interesting to note that at the three measuring times not a single QTL was assigned the L^*^ absolute value parameter, which is related to colour brightness (black/white gradient). The distribution of the a^*^ value led instead to identification of a QTL located on LG 16 (LOD: 3.85) when measured after cutting, and on LG 11 (LOD: 4.12) and 14 (LOD: 3.92) at the two stages (T_30_ and T_60_) of exposure to air (Figure 4 and Table 1). The change in LGs during the time-course was also detected for the b^*^ colorimetric parameter. Indeed, after cutting the QTL was identified on LG 9 (LOD: 4.0), while after 30 and 60 minutes another set of QTLs was instead targeted on LG 11 (LOD: 3.21), 13 (LOD: 3.93) and 14 (LOD: 3.39). LOD profile comparison of the three times for each colorimetric value revealed a shift in the set of chromosomes associated with the QTLs from the first assessment (immediately after cutting), as compared to fruit exposed to air. It is worth noting that the QTLs identified after cutting (T_0_), are mainly located on chromosomes 9 and 16, not being detected at any other location over the genome. These two QTLs should thus be more associated with flesh colour properties than flesh browning, hypothesis supported by the recent discovery of the gene set controlling red flesh in the apple and located respectively on these two chromosomes, such as *MdMYB10* [53,54] and *MdLAR* [55,56]. As browning is thought to be the intrinsic capacity of a fruit to change flesh colour after wounding, a delta (∆) value, calculated between two moments, would possibly be more effective in finding genomic regions involved in the control of browning development. When ∆ values (as well as the variation expressed as the percentage variation between T_30_-T_0_ and T_60_-T_0_) for the three colorimetric indexes (L^*^, a^*^ and b^*^) were considered in the computation, two linkage groups, LG 10 and LG 11, were mainly shown to be involved, spanning from a minimum LOD value of 3.08 (26.1 % of expressed variance) to a maximum of 4.68 (36.8%; Table 1). The last group of targeted QTLs was instead located on chromosome 14, and was associated with the ∆ value for a^*^ and b^*^ between T_60_ and T_30_, the absolute value for b^*^ at T_30_ and T_60_ and for a^*^ at T_60_, with LOD values from 3.39 (28.3%) to 4.99 (38.6%). Of the chromosomes identified in this survey considered to be relevant in the control of the flesh browning, chromosomes 10, 11 and 14 are the most significant, due to the presence of several QTLs in clusters, associated with different colour parameters, whereas the other three (9, 13 and 16) are represented by only one QTL each. The QTL-LOD profile defined for LG 11 also suggested the presence of two intervals associated with fruit flesh browning, one located at 25 cM and one below, at approximately 50 cM from the top of the linkage group. It is moreover worth noting that interesting QTLs associated with the concentration of Ascorbic Acid (AsA) and its oxidized form Dehydroascorbate (DHA), playing an important role in control of fruit flesh browning, were recently mapped [57] on chromosomes 10 (containing the *Md-PPO* gene) and 11. Indeed, in the apple the concentration of DHA was correlated to susceptibility to flesh browning [20], while in pears the tendency to develop internal browning was linked to a decreased concentration of AsA [58]. This finding, together with the results discussed here, suggests that chromosome 10 is the best candidate for regulation of the flesh browning phenotype, due to the simultaneous presence of genes responsible for its occurrence as well as its prevention. 

**Table 1:** Summary of QTLs associated with flesh browning identified in POP_1.

***Trait***	***LG***	***LOD***	***Marker***	***%Exp***	***mu_ac***	***mu_ad***	***mu_bc***	***mu_bd***
a*_T0	16	3.85	GDsnp00047	31.4	1.22	46.97	1.42	2.43
b*_T0	9	4	GDsnp00803	32.4	10.71	10.51	12.32	10.39
a*_T30	11	4.12	GDsnp01912	33.2	2.59	4.12	3.21	3.36
b*_T30	11	3.21	GDsnp00225	27	13.74	15.91	13.77	14.47
	13	3.93	CH01B12	31.9	16.54	13.68	14.14	14.29
a*_T60	11	3.52	GDsnp00418	29.2	2.94	4.79	3.56	3.8
	14	3.39	GDsnp00062	28.3	3.67	3.09	5.03	3.69
b*_T60	14	3.96	GDsnp01888	32.2	15.32	14.04	17.13	15.29
L_T30-T0	10	3.96	GDsnp02009	32.1	-3.78	-2.21	-2.42	-3.4
	11	4.68	GDsnp00240	36.8	-2.18	-4.01	-2.17	-2.88
a_T30-T0	10	3.08	GDsnp02183	26.1	2.22	1.28	1.52	2.32
	11	4.17	GDsnp00240	33.6	1.22	2.41	1.54	1.73
b_T30-T0	11	3.42	GDsnp00037	28.5	2.47	4.23	3.34	3.42
L_T60-T0	10	4.04	GDsnp02183	32.7	-4.78	-2.67	-3.29	-4.19
a_T60-T0	10	3.69	GDsnp02183	30.3	2.83	1.59	1.95	2.69
	11	4.39	GDsnp00240	35	1.57	2.94	1.87	2.24
L_T60-T30	14	4.04	GDsnp00165	32.7	-0.06	-0.75	-1.72	-0.47
a_T60-T30	14	4.99	GDsnp00165	38.6	0.35	0.33	0.91	0.32
L_T30-T0%	10	4.2	GDsnp02009	33.7	-5.23	-2.91	-3.22	-4.57
a_T30-T0%	11	3.39	GDsnp00037	28.3	95.06	201.69	124.7	119.44
L_T60-T0%	10	3.93	GDsnp02183	32	-6.47	-3.62	-4.4	-5.58
a_T60-T0%	11	3.39	GDsnp00240	28.3	119.88	242.25	147.89	151.07

The trait, linkage group (LG), maximum LOD value identified within the interval, closest marker to the LOD peak, percentage of expressed phenotypic variance (% Exp) and estimated mean of distribution for the four possible genotypes (mu_ac, mu_ad, mu_bc and mu_bd) are given for each QTL .

Finally, for better understanding the genetic control of flesh browning, the genomic intervals identified in this work were anchored on the genome assembly of ‘Golden Delicious’, in order to allow in silico annotation. Of the several genes identified over the six targeted chromosomes (Table S2), it is worth highlighting 52 elements (Table 2) mainly involved in cell wall metabolism and secondary metabolites (polyphenols [60-62]). In the first category, three gene families known to participate in cell wall disassembly were retrieved, namely pectinesterase, pectin lyase and polygalacturonase. Furthermore, the fifteen genes were mainly located on chromosomes 10 and 16, which are known to be hot spot regions for control of fruit texture physiology [25]. In particular, the presence of a polygalacturonase gene responsible for the texture variability observed in apple [48,51] has recently been validated on chromosome 10. The collocation of genes encoding cell wall degrading enzymes and QTLs discovered for fruit flesh browning has already been described in peach [59], where two browning QTLs co-mapped with candidates annotated as pectate lyase and expansin genes. The simultaneous presence of these two categories in the QTL intervals discovered for flesh browning can be explained by the fact that disassembly of internal cellular structure compartmentation is needed for flesh browning to occur. In this scenario, we can surmise the involvement of this class of genes to promote this process, facilitating the interplay between the polyphenol oxidase (stored in the plastid) and its phenolic substrate (stored in the vacuole). 

**Table 2:** Characterisation of 52 elements belonging to 16 categories mainly involved in cell wall and polyphenol metabolism.

**Gene family**	**Number**	**LG_9**	**LG_10**	**LG_11**	**LG_13**	**LG_14**	**LG_16**
Glycosyltransferase	10	3	1	4	1	1	0
pectinesterase	9	2	0	0	4	0	3
Cellulose synthase	5	0	4	0	0	0	1
Flavonoid o-methyltransferase	5	0	5	0	0	0	0
Polygalacturonase	5	0	1	0	1	0	3
Caffeic acid 3-O-methyltransferase	5	0	0	0	0	5	0
Chalcone synthase	2	2	0	0	0	0	0
Glucosyltransferase	2	0	0	2	0	0	0
Leucoanthocyanidin dioxygenase	2	0	0	0	2	0	0
flavonoid 3-o-glucosyltransferase	1	0	1	0	0	0	0
Isoflavone reductase	1	0	0	0	1	0	0
Trans-cinnamate 4-monooxygenase	1	0	0	0	1	0	0
Polyphenol oxidase	1	0	0	0	1	0	0
Cellulase	1	0	0	0	0	0	1
Isoflavonoid regulator	1	0	0	0	0	0	1
Pectin lyase	1	0	0	0	0	0	1

The relative number and chromosome locations are given for each class. This list is a specific selection of the complete QTL annotation given in Table S2.

Finally, another *PPO* element was newly discovered on chromosome 13, not previously targeted during in silico genome analysis (because of its poor sequence similarity with L29450), increasing the final number of *PPO* genes present in the apple genome to eleven.

### Md-PPO haplotype validation

**Figure 5:**
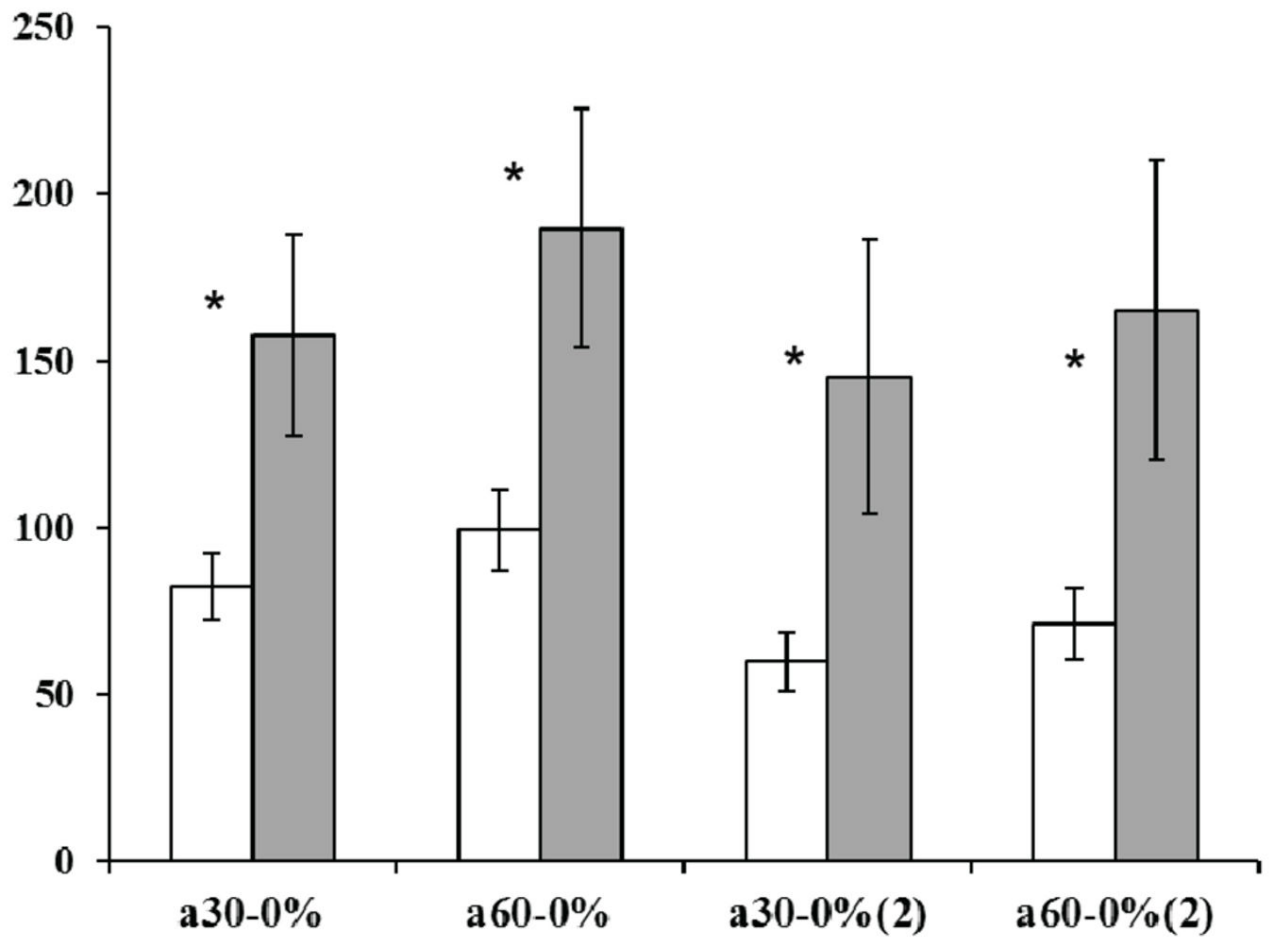
Md-PPO haplotype validation in the POP_2 progeny. White bars represent individuals characterised by a heterozygous haplotype (“np”), while the grey bars show the homozygous category (“nn”). The standard error is reported in each bar. On the x-axis, 30-0 and 60-0 are the percentage variation of ∆ a^*^ calculated between T_30_-T_0_ and T_60_-T_0_ respectively (2). indicates the ∆ value calculated after two months’ cold storage. As for the analysis carried out for the POP_1, five apples (biological replicates) were assessed for each genotypes, and for each fruit the flesh browning was measured of the two halves (technical replicates). Asterisks show statistically significant comparison based on the LSD-ANOVA test (*P*-value ≤ 0.05).

The effect of the Md-PPO haplotype (defined by the two SNPs targeted within the MDP0000699845 full-length) was further validated in POP_2 (‘Golden Delicious’ x ‘Braeburn’) assessed at two specific ripening stages, at harvest and after two months’ cold storage, in order to evaluate the effect of low storage temperature on flesh browning development. Marker validation was performed by grouping the seedlings into two classes according to their haplotypes (given as “np” and “nn” in Figure 5), which showed significant differences for ∆ a^*^ at T_30_-T_0_ and T_60_-T_0_ based on the LSD-ANOVA test (*P*value ≤ 0.05). For the two ripening stages (harvest and after two months’ cold storage) both parameters were statistically significant, with the heterozygous haplotype associated with a reduced variation in flesh colour, thus less prone to developing fruit flesh browning after cutting. These results also suggest that the colour change observed during flesh browning can be mostly attributed to a variation in the a^*^ parameter. This data is also in agreement with observations of significant changes in this colour index between T_0_ and T_30_ for the four apple cultivars, suggesting that fruit browning develops rapidly in the first 30 minutes (Figure S1). It is also interesting to note the differences observed during postharvest storage (2 months’ cold storage). The association observed between the rate of flesh browning and the Md-PPO haplotype was indeed confirmed after storage, but with a reduction of about 27% and 10% for the “np” and “nn” classes of seedling respectively. A reduced polyphenol content during cold storage [63] may reduce substrate availability for PPOs, resulting in a lower flesh browning rate. The difference observed between harvest and two months after storage was also confirmed by the *P*-values. At harvest, in fact, the value computed between the two seedling classes was more significant (*P*-value: 0.04) than the one calculated after storage (*P*-value: 0.05).

### Expression profiling and polyphenolic characterisation during the development of fruit flesh browning in apple

**Figure 6:**
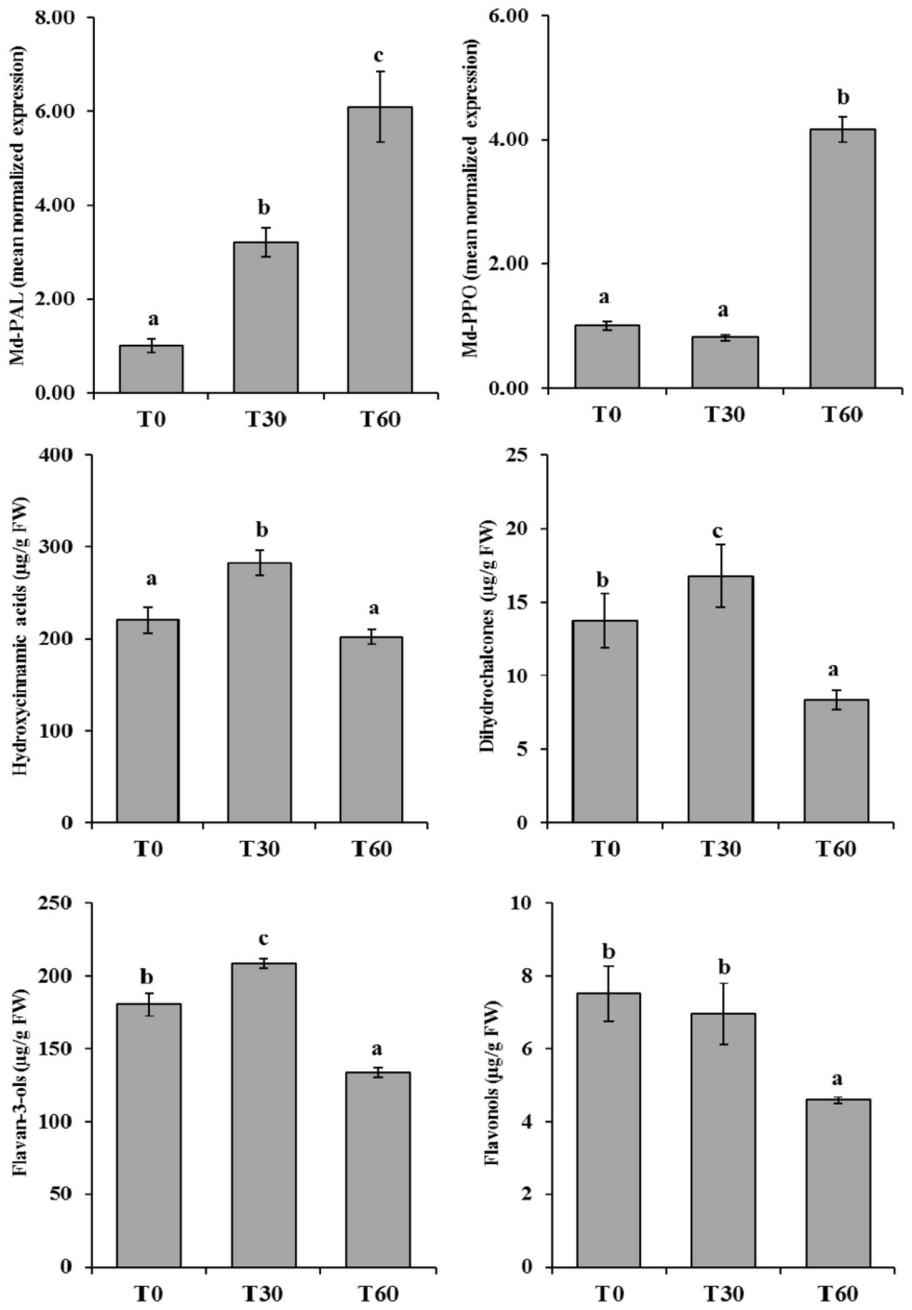
Expression profile of the two genes involved in the polyphenolic pathway, *PAL* (*Md-PAL*, panel a) and *PPO* (*Md-PPO*, panel b). In both graphs the y axis shows the mean normalised expression, graphing the three samples selected to monitor flesh browning (T_0_, T_30_ and T_60_). Over the same time-course, the accumulation of the four main polyphenolic compounds, namely hydroxycinnamic acid (c), dihydrochalcones (d), flavan-3-ols (e) and flavonols (f), is shown below in the figure. The amount of these compounds is plotted on the y axis and expressed as μg/g of fresh weight (FW). Polyphenolic profiling was performed analyzing four replicates/experimental time. For each bar the standard error is also reported. Samples significantly different are shown using different letters following a LSD-ANOVA test (*P*-value ≤ 0.05).

To explain the physiological regulation leading to the occurrence of fruit flesh browning in more detail, two main genes were considered, *Md-*PAL (phenylalanine ammonia lyase) and *Md-PPO*. The first gene, *Md-PAL*, is responsible for the biotransformation of L-phenyalanine to ammonia and trans-cinnamic acid, which is the first step in the biosynthetic pathway of polyphenol compounds [64,65]. The second, *Md-PPO*, is the same element investigated here and genetically associated with the flesh browning observed in the two populations. The expression profile of these two genes over the time-course showed distinct temporal activation, also in agreement with their respective positions along the polyphenol cascade (Figure 1). *Md-PAL*, which is located upstream in this pathway, was progressively expressed throughout the time-course, already showing increased mRNA accumulation after 30 minutes’ (T_30_) exposure to air (Figure 6a), consistent with the different accumulation of the main polyphenol classes. At T_30_ a general accumulation was indeed observed for hydroxycinnamic acid, dihydrochalcone and flavan-3-ol classes (Figure 6 c, d, e), while an almost unchanged situation was observed for flavonols (Figure 6f). It is worth noting that individual compounds showed the same trend as their respective phenolic groups, as indicated in Table S3. Increased production of these compounds by wounding has already been observed in lettuce [66], but never clearly examined in apple. This enhanced production, coordinated by the activation of *Md-PAL* expression, can be considered a response of the defence mechanism (antioxidant protection) as well as signalling [67,68]. In this scenario we can hypothesize that PPO enzyme is eventually synthesized to oxidize the amount of polyphenols produced after wounding, theory supported by the late expression observed for *Md-PPO*. This gene, located downstream in this pathway, indeed showed basal and consistent expression between T_0_ and T_30_, which was crucial in the development of flesh browning, while at T_60_ its transcript accumulation increased by about 5 fold (Figure 6b). 

These functional dynamics were also validated by metabolite screening of polyphenol characterisation. At T_60_ the four main polyphenol classes, described in Figure 6 (c, d, e, f), showed an average concentration decrease of about 1.5-2 fold. The browning colouration occurring in the first 30 minutes may be possibly caused by PPO enzymes already available and stored in the plastid. After this activation, the wounded organs stimulate higher production of polyphenols as a defence signal, which is then maintained by a feed-back controlling mechanism, through the production of additional PPO enzymes devoted to their oxidation. In this system the fruit can regulate the signalling triggered by the wounding events, while the flesh browning appearing immediately after cutting seems to be more related to the initial genetically determined amount of PPO enzyme, together with its substrates.

## Conclusion

This work offers new insight, shedding light on the genetic regulation of apple flesh browning. This flesh browning, which seriously decreases the quality of the final fruit in minimally processed fruit, seems to be related to a genetically determined accumulation of the PPO enzyme, which can differ greatly in the various apple cultivars. This theory is supported by the fact that *Md-PPO* gene is highly activated only in the last phase of the time-course designed here, while browning occurs much earlier, corresponding to the increased transcription of another gene, *Md-PAL*, known to stimulate the entire polyphenolic cascade. *Md-PPO* was then thought to be activated more to regulate the polyphenolic signalling system. However, this gene showed two distinct haplotypes, which may be responsible for the initial enzymatic amount stored in the cell (before wounding), responsible for the extent of final browning.

The availability of a marker associated with this phenomenon may represent a valid alternative to destructive methods for the selection of low browning accession, suitable for improving the quality of minimally processed apples.

## Supporting Information

Figure S1 **Fruit flesh browning evolution in the four parental apple cultivars, ‘Fuji’(i), ‘Pink Lady’ (ii), ‘Golden Delicious’ (iii) and ‘Braeburn’ (iv).** For each variety the three panels are a_T_0_ (after cutting), b_T_30_ (after 30 minutes) and c_T_60_ (after 60 minutes). The histograms below each panel give the digital colour measurements obtained by the colorimeter and expressed as L*, a* and b*. Also for the four parental cultivars, five apples were assessed for each experimental time (T_0,_ T_30_ and T_60_), representing the biological replicates, on which two colour measurements were performed on the two sides of a cut apple (technical replicates). The letters given show the statistical significance following to the LSD-ANOVA test (*P*-value ≤ 0.05). In slide “v” the difference between the two parental cultivars for L*, a* and b* values measured at T_0_, T_30_ and T_60_ is shown for each progeny. Statistically significant differences (P value ≤ 0.05) are highlighted with asterisks. The four parents are indicated as follows: ‘Fuji’ (green), ‘Pink Lady’ (pink), ‘Golden Delicious’ (blue) and ‘Braeburn’ (red). For each bar the standard error is also visualized. Below each histogram the actual value is reported.
(PPT)

Figure S2 **Allelic polymorphism and segregation of the microsatellite marker MdPPO_SSR_ch5e.** In the figure the first two rows refer to ‘Golden Delicious’ and ‘Braeburn’.
(PPT)

Figure S3 **Allelic polymorphism and segregation of the microsatellite marker MdPPO_SSR_ch10d.** In the figure the first two rows refer to ‘Braeburn’ and ‘Golden Delicious’.
(PPT)

Figure S4 **POP_1 (‘Fuji x Pink Lady’) genetic map.** The red text highlights the genetic position of the two SSR markers, respectively positioned on chromosome 5 (MdPPO_SSR_ch5e) and 10 (MdPPO_SSR_ch10d).
(PPT)

Figure S5 **POP_2 (‘Golden Delicious x Braeburn’) genetic map.** The red text highlights the genetic position of the two SSR markers, respectively positioned on chromosome 5 (MdPPO_SSR_ch5e) and 10 (MdPPO_SSR_ch10d).
(PPT)

Figure S6 **Nucleotide and aminoacid sequence of *Md-PPO*.** Section “a” of the figure shows the Md-PPO nucleotide sequence of ‘Golden Delicious’ (>PPO_GD; accession number: KF561111) and ‘Braeburn’ (>PPO_Br; accession number: KF561112). In the two sequences the two SNPs are highlighted in bold black text. Section “b” shows the alignment of the predicted aminoacids in ‘Golden Delicious’ (GD_prot) and Braeburn (Br_prot). The aminoacid substitution conferred by SNP_170_ is shown in bold black text.
(DOC)

Table S1 **List of microsatellite marker primers designed on the contigs, related to the ten targeted PPO genes.** The name, primer sequences, number of the contig and chromosome are given for each marker.
(DOC)

Table S2 **Annotation of the gene underlying the QTL intervals identified on chromosomes 9, 10, 11, 13, 14 and 16.**
(XLS)

Table S3 **Trend for each individual phenolic compound.** The mean concentration and respective standard deviation are given for each phenolic compound.
(XLS)
