# Correction: A Multidisciplinary Approach Providing New Insight into Fruit Flesh Browning Physiology in Apple (*Malus x domestica* Borkh.)

**DOI:** 10.1371/annotation/72dfe3df-4cfd-49a8-93db-979bdd8794c3

**Published:** 2014-01-06

**Authors:** Mario Di Guardo, Alice Tadiello, Brian Farneti, Giorgia Lorenz, Domenico Masuero, Urska Vrhovsek, Guglielmo Costa, Riccardo Velasco, Fabrizio Costa

The affiliation for the fifth and sixth authors is incorrect. Domenico Masuero and Urska Vrhovsek are not affiliated with institution number 1, but rather with the following institution: Department of Food Quality and Nutrition, Research and Innovation Centre, Fondazione Edmund Mach, San Michele all’Adige (Trento), Italy. 

