# Supplementary material for: A Multidisciplinary Approach Providing New Insight into Fruit Flesh Browning Physiology in Apple (Malus x domestica Borkh.)
Source: PLoS One. 2013 Oct 18;8(10):e78004. doi: 10.1371/journal.pone.0078004 (PMC3799748; doi:10.1371/journal.pone.0078004)
Supplement: Figure S1 — Fruit flesh browning evolution in the four parental apple cultivars, ‘Fuji’(i), ‘Pink Lady’ (ii), ‘Golden Delicious’ (iii) and ‘Braeburn’ (iv). For each variety the three panels are a_T0 (after cutting), b_T30 (after 30 minutes) and c_T60 (after 60 minutes). The histograms below each panel give the digital colour measurements obtained by the colorimeter and expressed as L*, a* and b*. Also for the four parental cultivars, five apples were assessed for each experimental time (T0, T30 and T60), representing the biological replicates, on which two colour measurements were performed on the two sides of a cut apple (technical replicates). The letters given show the statistical significance following to the LSD-ANOVA test (P-value ≤ 0.05). In slide “v” the difference between the two parental cultivars for L*, a* and b* values measured at T0, T30 and T60 is shown for each progeny. Statistically significant differences (P value ≤ 0.05) are highlighted with asterisks. The four parents are indicated as follows: ‘Fuji’ (green), ‘Pink Lady’ (pink), ‘Golden Delicious’ (blue) and ‘Braeburn’ (red). For each bar the standard error is also visualized. Below each histogram the actual value is reported. (PPT) [file pone.0078004.s001.ppt]

## Slide 1
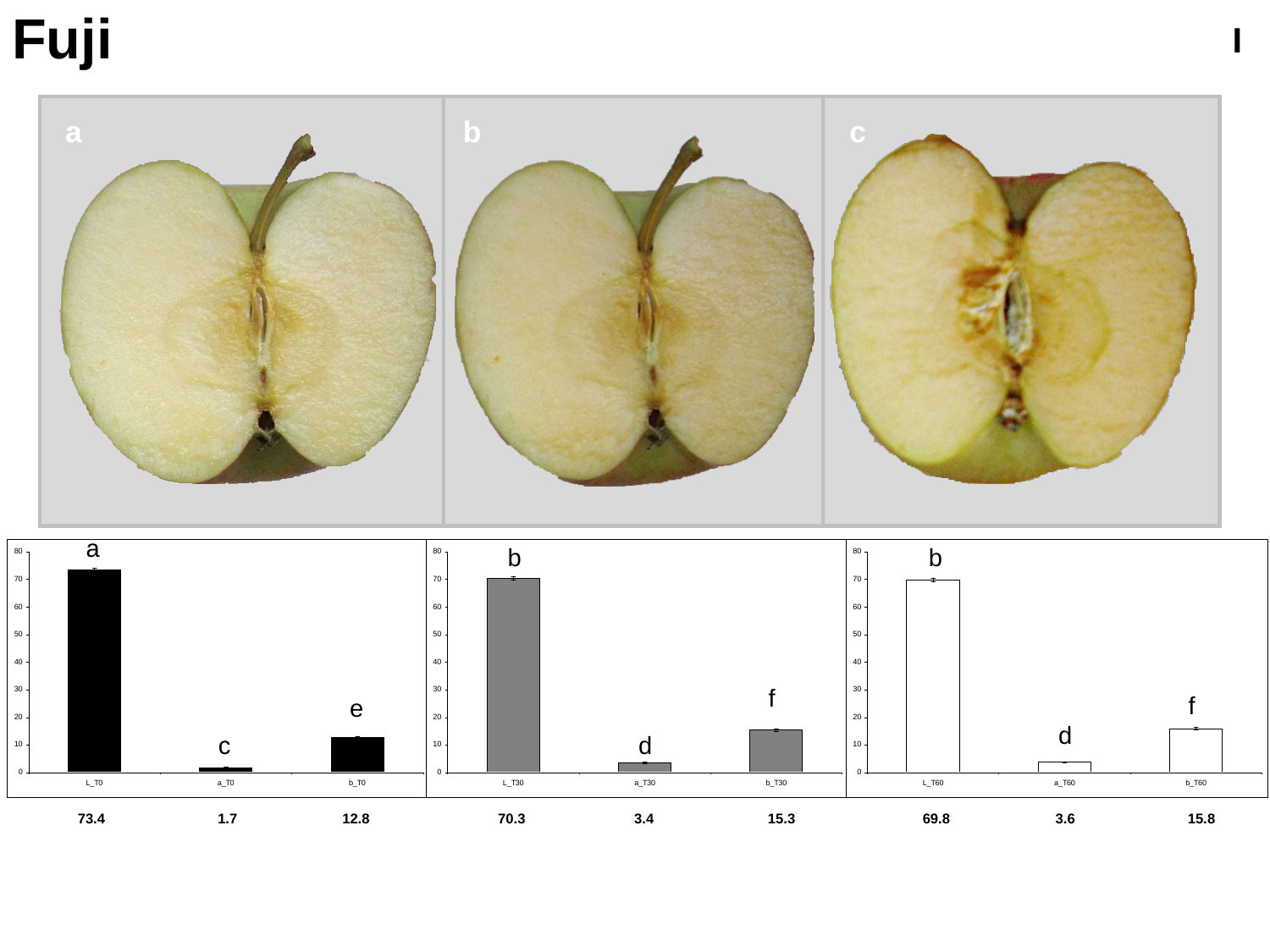

Fuji
I
b
c
a
a
b
c
a
b
b
f
f
e
d
c
d
1.7
12.8
70.3
3.4
69.8
3.6
15.8
73.4
15.3

## Slide 2
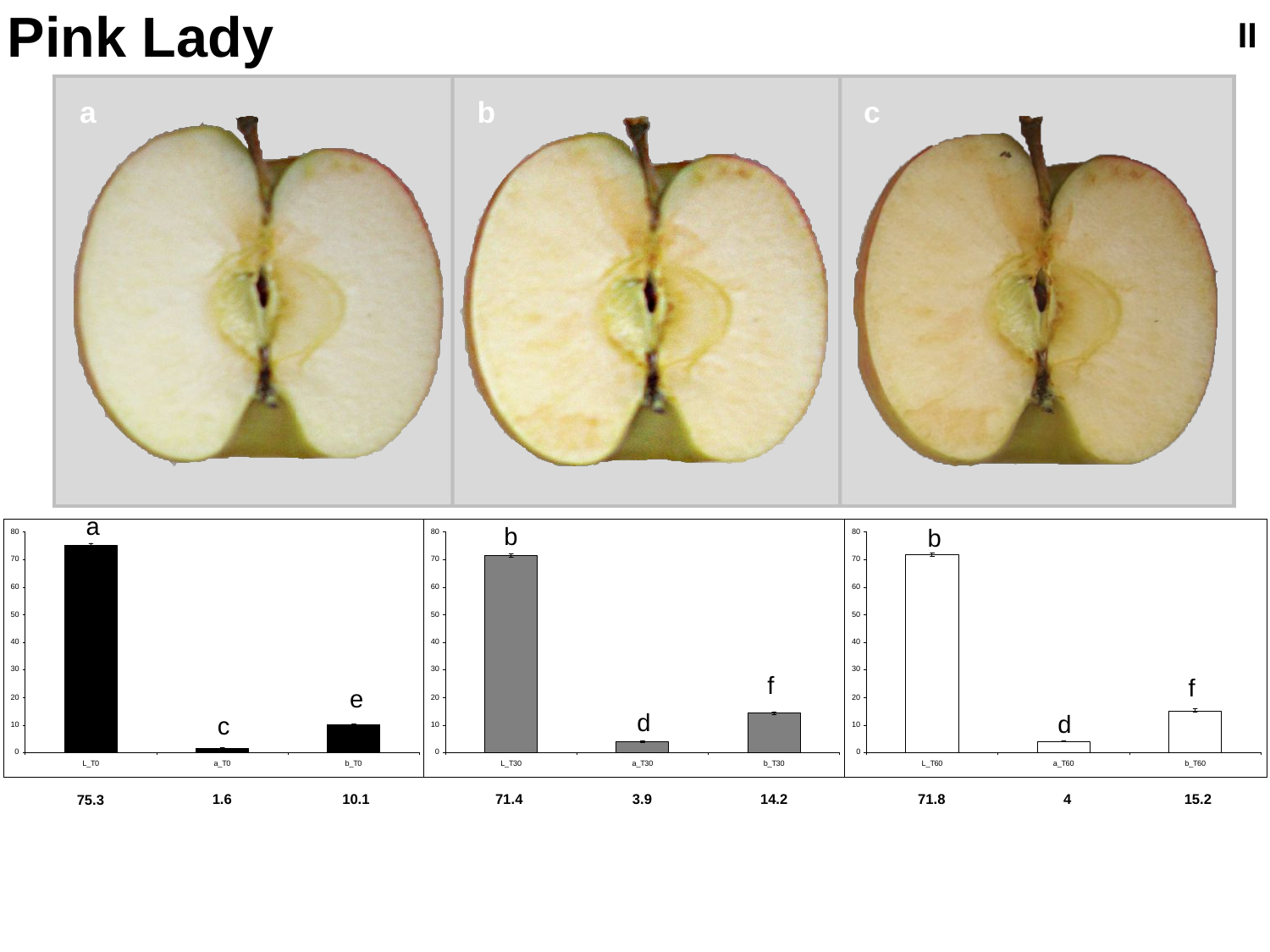

Pink Lady
II
b
c
a
a
b
b
f
f
e
d
d
c
1.6
10.1
71.4
3.9
14.2
71.8
4
15.2
75.3

## Slide 3
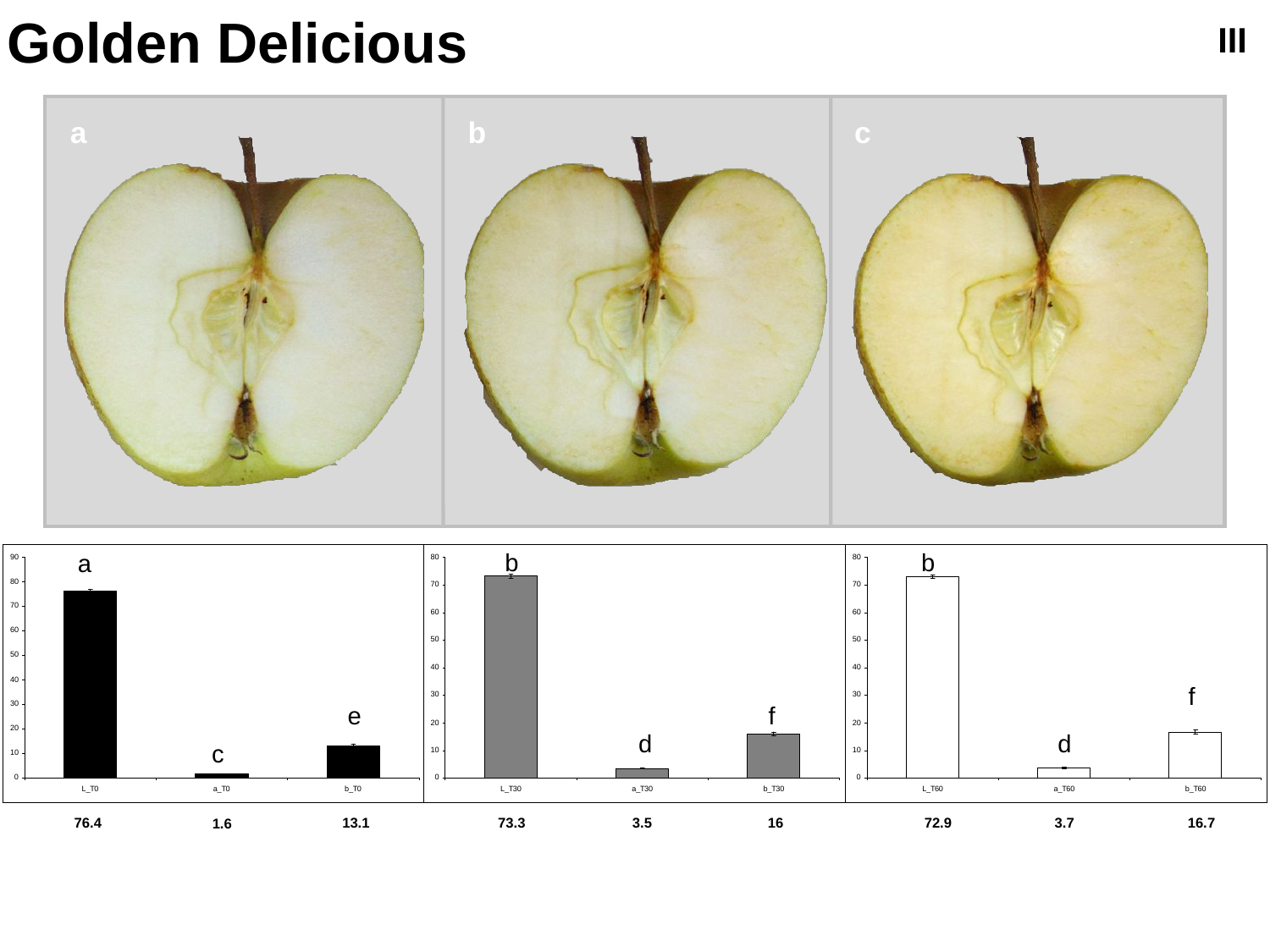

Golden Delicious
III
b
c
a
b
b
a
f
e
f
d
d
c
76.4
13.1
73.3
3.5
16
72.9
3.7
16.7
1.6

## Slide 4
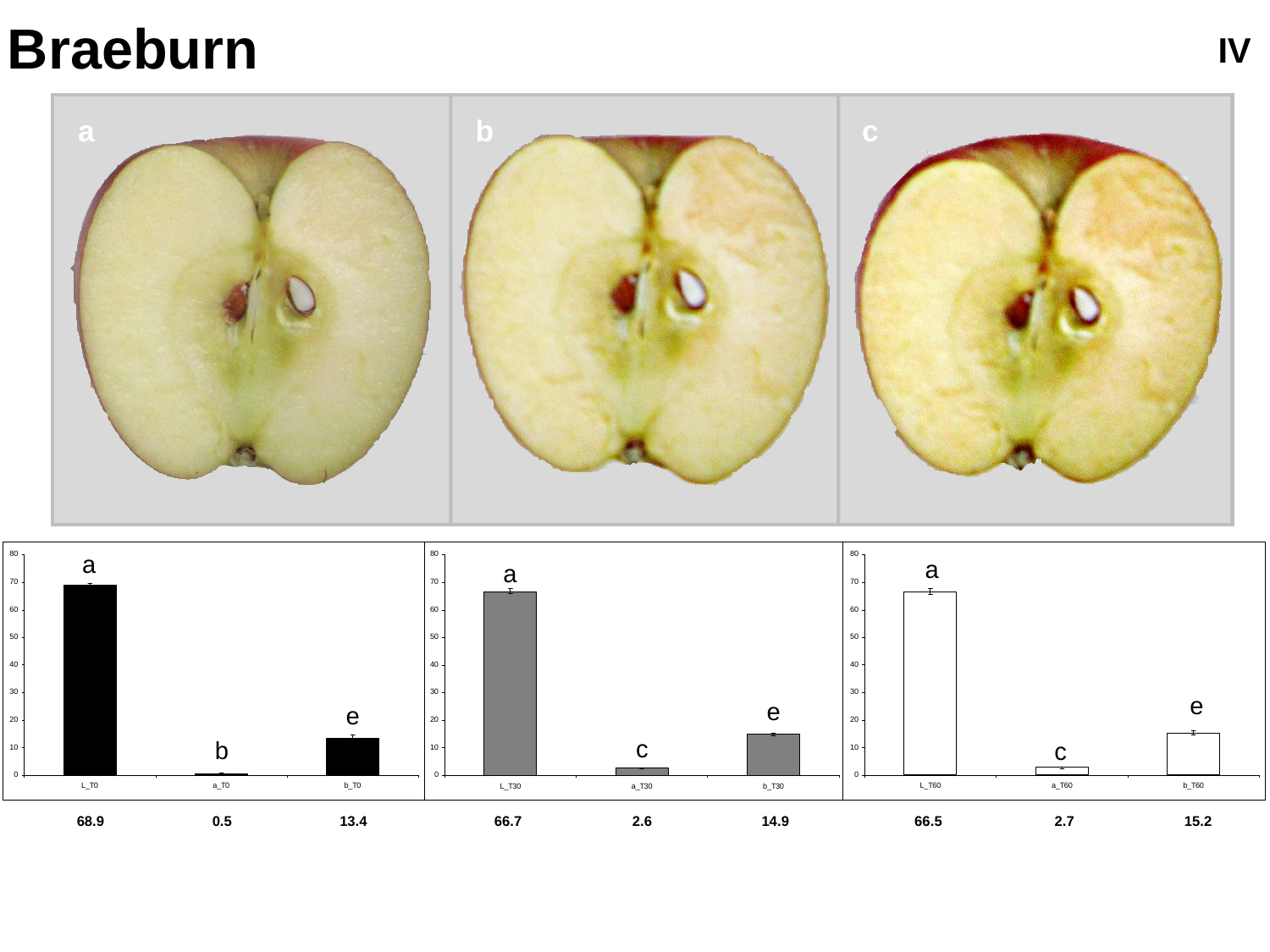

Braeburn
IV
b
c
a
a
a
a
e
e
e
c
b
c
68.9
0.5
13.4
66.7
2.6
14.9
66.5
2.7
15.2

## Slide 5
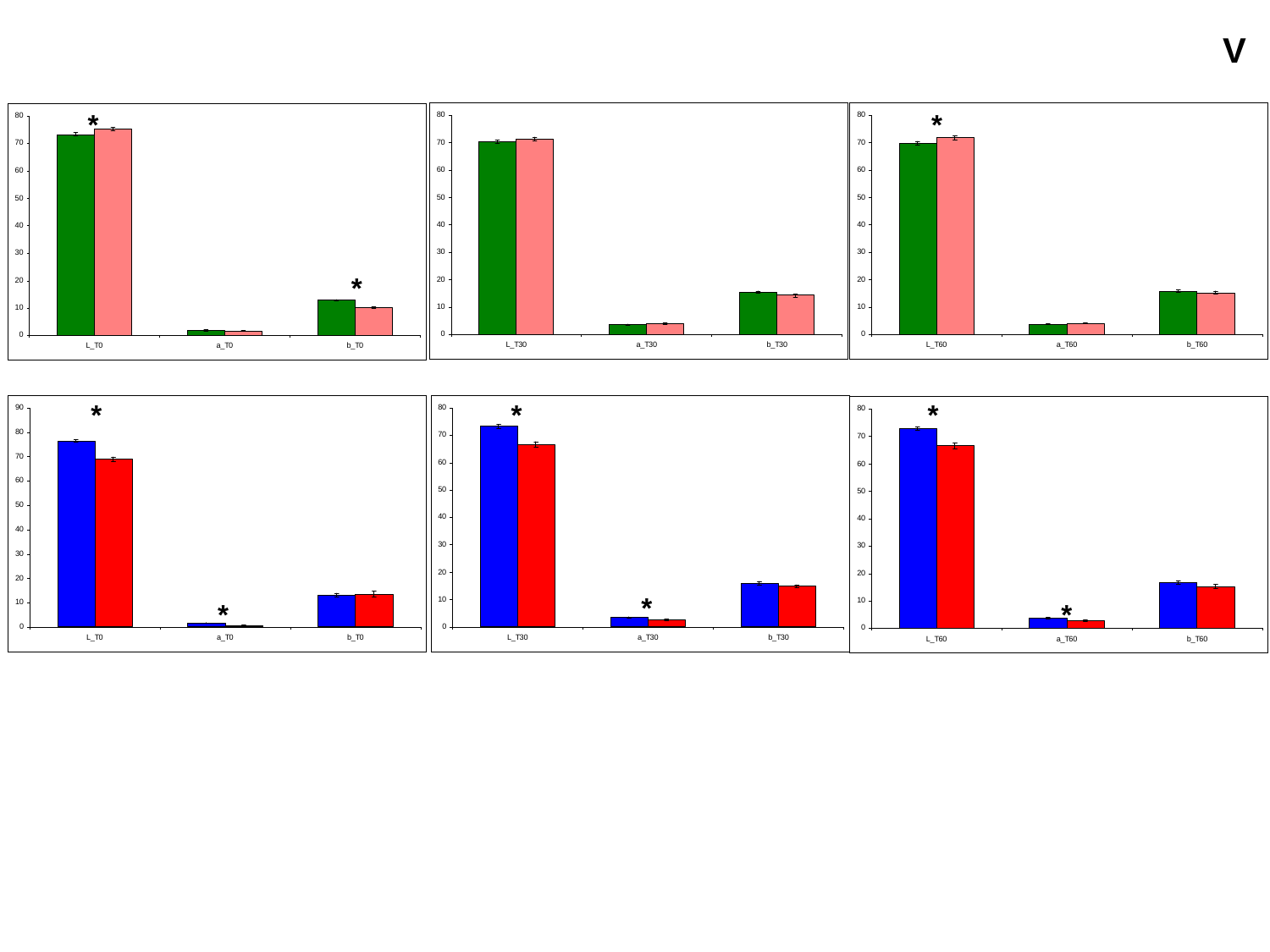

V
*
*
*
*
*
*
*
*
*
