# Supplementary figures and images for: A Multidisciplinary Approach Providing New Insight into Fruit Flesh Browning Physiology in Apple (Malus x domestica Borkh.)
Source: PLoS One. 2013 Oct 18;8(10):e78004. doi: 10.1371/journal.pone.0078004 (PMC3799748; doi:10.1371/journal.pone.0078004)

## Slide 1
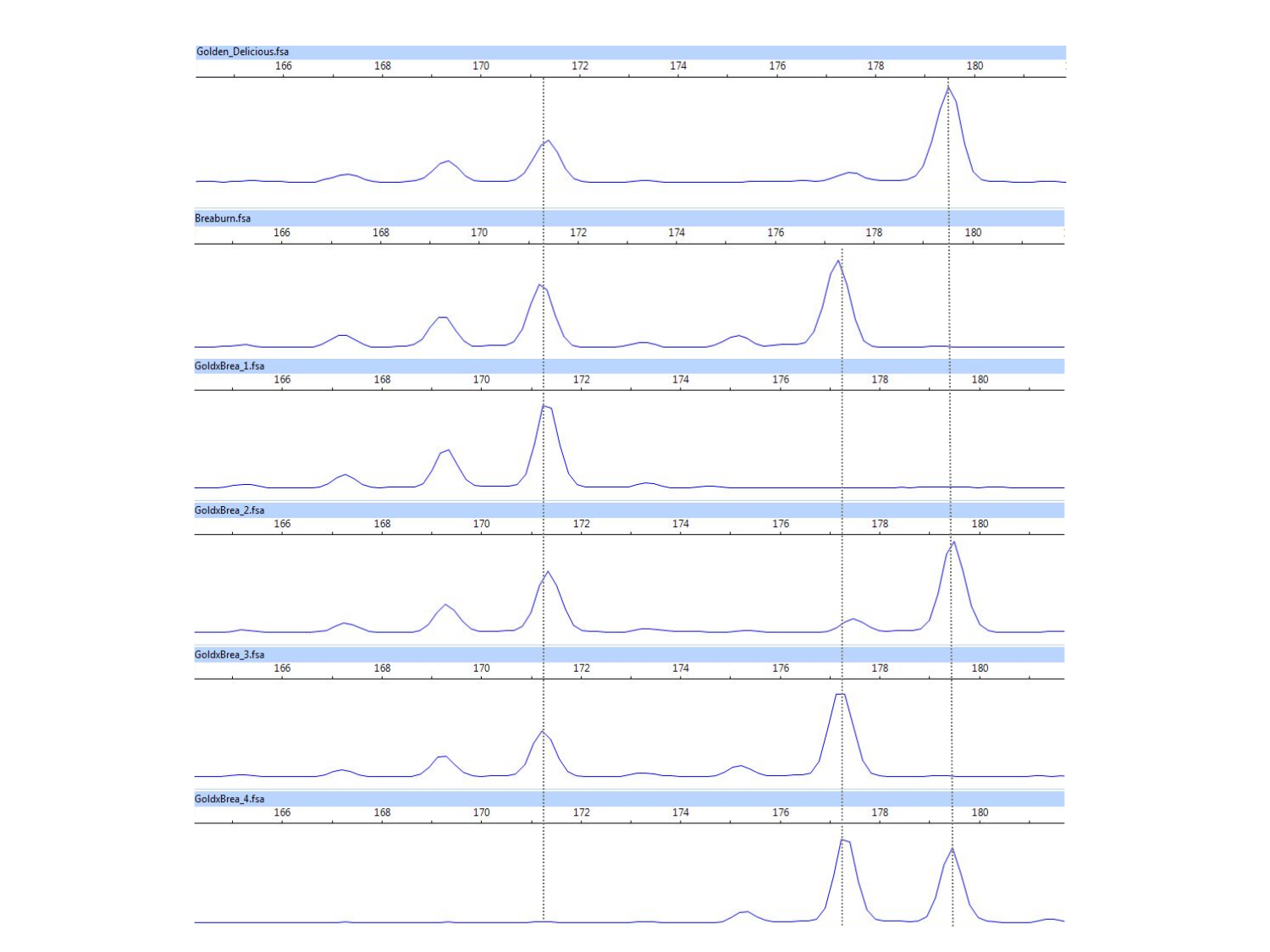

Supplement: Figure S2 — Allelic polymorphism and segregation of the microsatellite marker MdPPO_SSR_ch5e. In the figure the first two rows refer to ‘Golden Delicious’ and ‘Braeburn’. (PPT) [file pone.0078004.s002.ppt]

## Slide 1
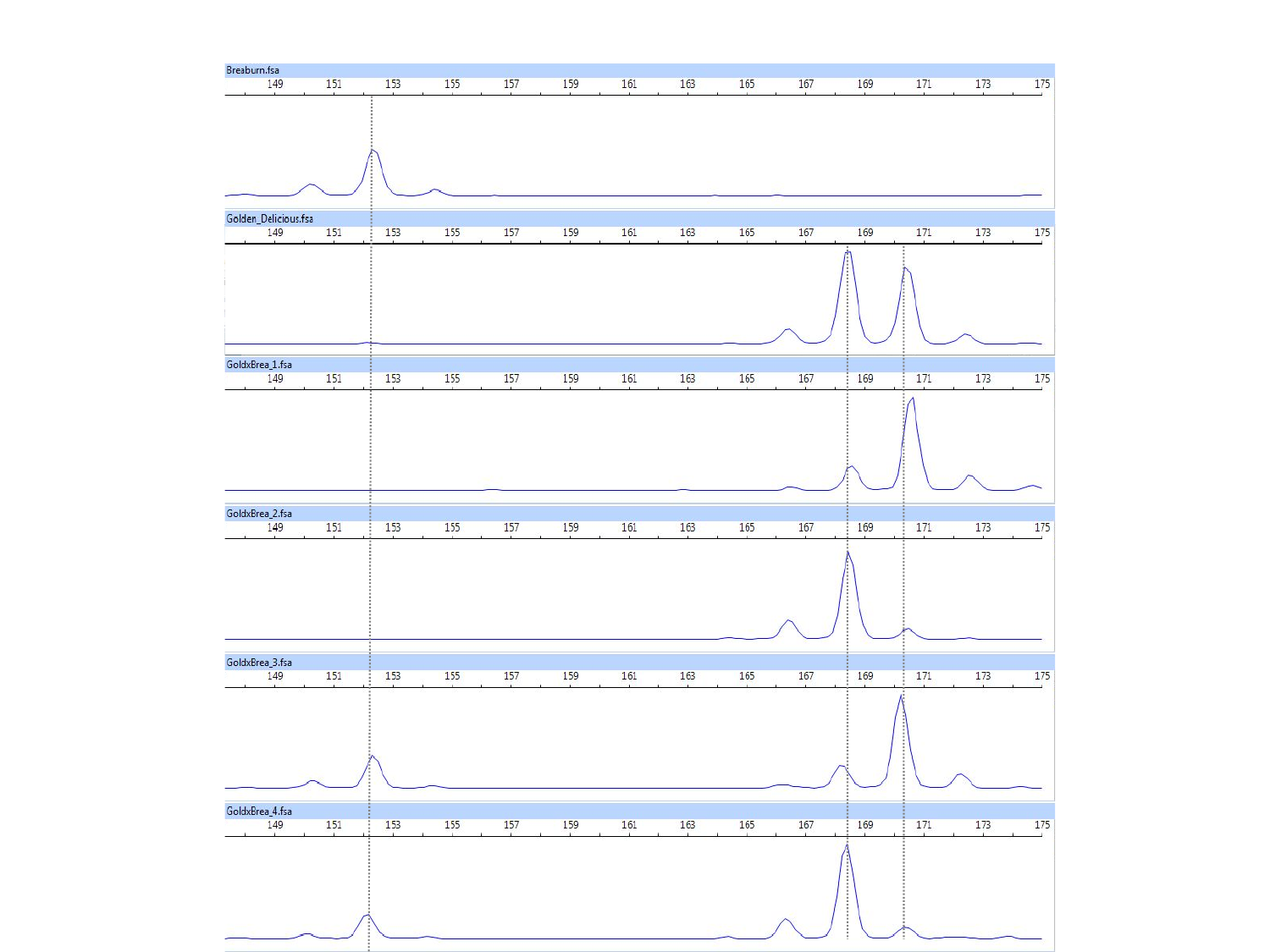

Supplement: Figure S3 — Allelic polymorphism and segregation of the microsatellite marker MdPPO_SSR_ch10d. In the figure the first two rows refer to ‘Braeburn’ and ‘Golden Delicious’. (PPT) [file pone.0078004.s003.ppt]

## Slide 1
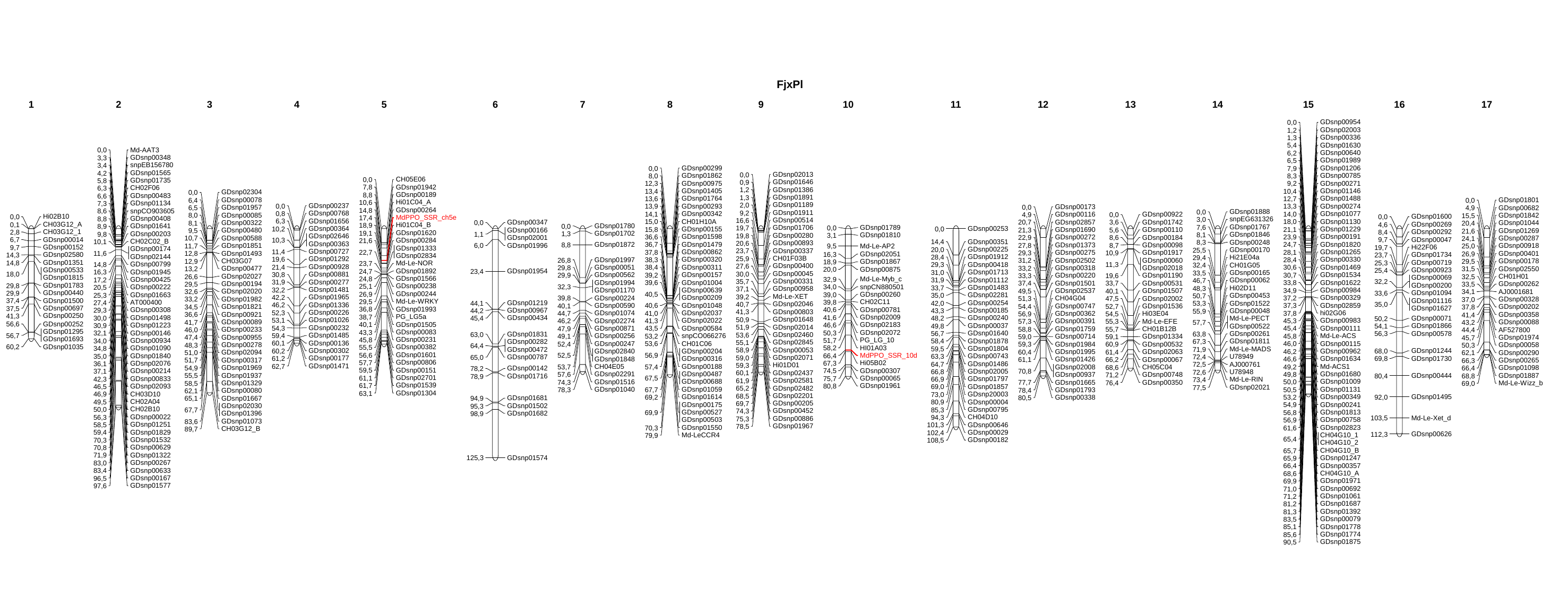

Supplement: Figure S4 — POP_1 (‘Fuji x Pink Lady’) genetic map. The red text highlights the genetic position of the two SSR markers, respectively positioned on chromosome 5 (MdPPO_SSR_ch5e) and 10 (MdPPO_SSR_ch10d). (PPT) [file pone.0078004.s004.ppt]
