# Supplementary material for: A Multidisciplinary Approach Providing New Insight into Fruit Flesh Browning Physiology in Apple (Malus x domestica Borkh.)
Source: PLoS One. 2013 Oct 18;8(10):e78004. doi: 10.1371/journal.pone.0078004 (PMC3799748; doi:10.1371/journal.pone.0078004)
Supplement: Figure S5 — POP_2 (‘Golden Delicious x Braeburn’) genetic map. The red text highlights the genetic position of the two SSR markers, respectively positioned on chromosome 5 (MdPPO_SSR_ch5e) and 10 (MdPPO_SSR_ch10d). (PPT) [file pone.0078004.s005.ppt]

## Slide 1
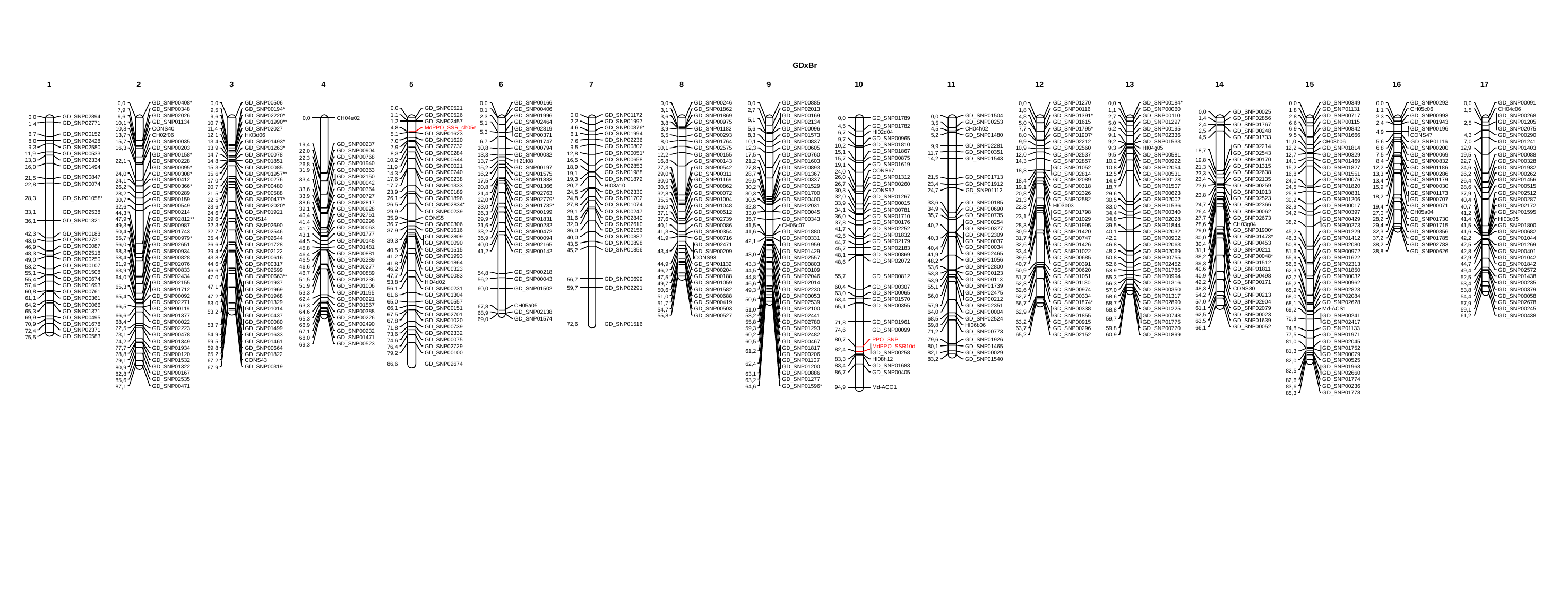

GDxBr
1
GD_SNP00408*
0,0
GD_SNP00348
7,9
GD_SNP02026
GD_SNP02894
9,6
0,0
GD_SNP01134
GD_SNP02771
10,1
1,4
10,8
CONS40
6,7
GD_SNP00152
13,7
CH02f06
8,0
GD_SNP02428
15,7
GD_SNP00035
9,3
GD_SNP02580
16,3
GD_SNP00203
11,9
GD_SNP00533
GD_SNP00158*
13,3
GD_SNP02334
22,1
GD_SNP00228
16,0
GD_SNP01494
GD_SNP00095*
24,0
GD_SNP00308*
GD_SNP00847
21,5
GD_SNP00412
24,1
GD_SNP00074
22,8
GD_SNP00366*
26,2
GD_SNP00289
28,2
28,3
GD_SNP01058*
GD_SNP00159
30,7
GD_SNP00549
32,6
33,1
GD_SNP02538
GD_SNP00214
44,3
47,9
GD_SNP02812**
GD_SNP01321
36,1
49,3
GD_SNP00987
50,4
GD_SNP01743
42,3
GD_SNP00183
55,7
GD_SNP00979*
GD_SNP02731
43,6
56,0
GD_SNP02651
GD_SNP00087
46,9
58,3
GD_SNP00934
GD_SNP02518
48,3
58,4
GD_SNP00828
GD_SNP00250
49,0
61,9
GD_SNP02076
GD_SNP00107
53,2
GD_SNP00833
GD_SNP01508
55,1
GD_SNP00674
55,4
57,4
GD_SNP01693
60,8
GD_SNP00761
61,1
GD_SNP00361
64,2
GD_SNP00066
65,3
GD_SNP01371
69,9
GD_SNP00495
70,9
GD_SNP01678
GD_SNP02371
72,4
GD_SNP00583
75,5
2
GD_SNP00506
0,0
GD_SNP00194*
9,5
GD_SNP02220*
9,6
GD_SNP01990**
10,7
11,4
GD_SNP02027
12,1
Hi03d06
13,4
GD_SNP01493*
13,9
GD_SNP01263*
14,7
GD_SNP00078
14,8
GD_SNP01851
15,3
GD_SNP00085
15,6
GD_SNP01957**
GD_SNP00276
17,0
GD_SNP00480
20,7
GD_SNP00588
21,5
GD_SNP00477*
22,5
GD_SNP02020*
23,6
GD_SNP01921
24,6
29,6
CONS14
32,3
GD_SNP02690
32,7
GD_SNP02546
35,4
GD_SNP02644
36,6
GD_SNP01728
39,4
GD_SNP02122
43,8
GD_SNP00616
44,6
GD_SNP00317
GD_SNP02599
63,9
46,6
GD_SNP02434
GD_SNP00663**
64,0
47,0
GD_SNP02155
GD_SNP01937
65,3
47,1
GD_SNP01712
GD_SNP01969
GD_SNP00092
GD_SNP01968
65,4
47,2
GD_SNP02271
GD_SNP01329
53,0
66,5
GD_SNP00119
GD_SNP01014
53,2
66,6
GD_SNP01377
GD_SNP00437
68,4
GD_SNP00022
GD_SNP00080
53,7
72,5
GD_SNP02223
GD_SNP01499
73,1
GD_SNP00478
54,9
GD_SNP01633
74,2
GD_SNP01349
59,5
GD_SNP01461
77,7
GD_SNP01934
78,8
GD_SNP00120
GD_SNP01532
79,1
GD_SNP01322
80,9
GD_SNP00167
82,8
GD_SNP02535
85,6
GD_SNP00471
87,1
3
4
0,0
GD_SNP00521
1,1
GD_SNP00526
0,0
CH04e02
1,2
GD_SNP02457
4,8
MdPPO_SSR_ch05e
GD_SNP01623
5,1
GD_SNP01620
7,0
GD_SNP00237
19,4
GD_SNP02732
7,9
GD_SNP00904
22,0
GD_SNP00284
8,3
GD_SNP00768
22,3
GD_SNP00544
10,2
GD_SNP01940
26,8
GD_SNP00021
11,9
31,9
GD_SNP00363
GD_SNP00740
14,3
GD_SNP02150
17,6
GD_SNP00238
33,4
GD_SNP00042
17,7
GD_SNP01333
33,6
GD_SNP00364
23,9
GD_SNP00189
33,9
GD_SNP00727
26,1
GD_SNP01896
38,6
GD_SNP02817
26,5
GD_SNP02834*
39,1
GD_SNP00928
29,9
GD_SNP00239
GD_SNP02751
40,4
35,9
CONS5
GD_SNP02296
41,4
36,7
GD_SNP00306
GD_SNP00063
41,7
GD_SNP01616
37,9
GD_SNP01777
43,1
GD_SNP00148
44,5
GD_SNP01481
45,8
GD_SNP00881
46,4
46,5
GD_SNP02289
46,6
GD_SNP00277
46,7
GD_SNP00889
51,5
GD_SNP01236
51,9
GD_SNP01006
53,3
GD_SNP01195
62,4
GD_SNP00221
GD_SNP01567
63,3
GD_SNP00388
64,6
GD_SNP00226
65,3
GD_SNP02490
66,9
GD_SNP00232
67,1
GD_SNP01471
68,0
GD_SNP00523
69,3
59,8
GD_SNP00664
65,2
GD_SNP01822
CONS43
67,2
GD_SNP00319
67,9
5
6
GD_SNP00166
0,0
GD_SNP00406
0,1
GD_SNP01996
2,3
GD_SNP02464
5,1
GD_SNP02819
5,3
GD_SNP00371
6,7
GD_SNP01747
10,8
GD_SNP00794
13,3
GD_SNP00082
13,7
Hi21f08
15,2
GD_SNP00197
16,2
GD_SNP01575
GD_SNP01883
17,5
GD_SNP01366
20,8
GD_SNP02763
21,4
GD_SNP02779*
22,0
GD_SNP01732*
23,0
GD_SNP00199
26,3
29,9
GD_SNP01831
31,6
GD_SNP00282
33,2
GD_SNP00472
GD_SNP02809
36,9
GD_SNP00094
39,3
GD_SNP00090
40,0
GD_SNP02165
GD_SNP01515
40,5
41,2
GD_SNP00142
GD_SNP01993
41,2
GD_SNP01864
41,8
46,2
GD_SNP00323
GD_SNP00218
54,8
47,7
GD_SNP00083
GD_SNP00043
56,2
53,8
Hi04d02
56,1
GD_SNP00231
60,0
GD_SNP01502
61,6
GD_SNP01304
65,0
GD_SNP00557
CH05a05
67,8
66,1
GD_SNP00151
GD_SNP02138
68,9
67,5
GD_SNP02701
GD_SNP01574
69,0
GD_SNP01020
67,8
GD_SNP00739
71,8
GD_SNP02332
73,6
GD_SNP00075
74,6
GD_SNP02729
76,4
GD_SNP00100
79,2
86,6
GD_SNP02674
7
GD_SNP00246
0,0
GD_SNP01862
3,1
0,0
GD_SNP01172
GD_SNP01869
3,6
2,2
GD_SNP01997
GD_SNP00975
3,8
4,6
GD_SNP00876*
3,9
GD_SNP01182
GD_SNP01994
6,1
6,5
GD_SNP00293
GD_SNP02236
7,6
8,0
GD_SNP01764
GD_SNP00802
9,5
10,1
GD_SNP02575
GD_SNP00051*
12,8
12,2
GD_SNP00155
GD_SNP00658
16,5
16,8
GD_SNP00143
GD_SNP02853
18,9
27,3
GD_SNP00542
GD_SNP01988
19,1
29,0
GD_SNP00311
19,3
GD_SNP01872
GD_SNP01169
30,0
20,7
HI03a10
GD_SNP00862
30,5
24,5
GD_SNP02330
GD_SNP00072
32,8
24,8
GD_SNP01702
GD_SNP01004
35,5
27,8
GD_SNP01074
GD_SNP01048
36,0
29,1
GD_SNP00247
GD_SNP00512
37,1
31,6
GD_SNP02840
37,6
GD_SNP02739
32,0
GD_SNP02610
40,1
GD_SNP00086
GD_SNP02156
36,0
41,3
GD_SNP00354
GD_SNP00887
40,0
41,9
GD_SNP00716
GD_SNP00898
43,5
GD_SNP02471
GD_SNP01856
45,2
43,4
GD_SNP00209
CONS93
44,9
GD_SNP01132
GD_SNP00204
46,2
GD_SNP00188
47,5
GD_SNP00699
56,7
GD_SNP01059
49,7
59,7
GD_SNP02291
GD_SNP01582
50,6
GD_SNP00688
51,0
GD_SNP00419
51,7
72,6
GD_SNP01516
8
9
GD_SNP00885
0,0
GD_SNP02013
2,7
GD_SNP00169
0,0
GD_SNP01789
5,1
GD_SNP02134
4,5
GD_SNP01782
5,6
GD_SNP00096
HI02d04
6,7
8,3
GD_SNP01573
GD_SNP00965
9,7
10,1
GD_SNP00837
GD_SNP01810
10,2
12,3
GD_SNP00605
17,5
GD_SNP00760
21,2
GD_SNP01603
27,8
GD_SNP00893
28,7
GD_SNP01367
GD_SNP00337
29,5
GD_SNP01529
30,2
GD_SNP01700
30,3
GD_SNP00400
30,5
GD_SNP02031
32,8
GD_SNP00045
33,0
35,7
GD_SNP00343
41,5
CH05c07
41,6
GD_SNP01880
GD_SNP00331
42,1
GD_SNP01959
GD_SNP01429
43,0
GD_SNP02557
43,3
GD_SNP00803
GD_SNP00109
44,5
GD_SNP02046
44,8
GD_SNP02014
46,6
GD_SNP02230
49,3
GD_SNP00053
50,6
GD_SNP02539
GD_SNP00503
GD_SNP02100
54,7
51,0
55,8
GD_SNP00527
53,2
GD_SNP02441
55,8
GD_SNP02780
59,3
GD_SNP01293
60,2
GD_SNP02482
60,5
GD_SNP00467
GD_SNP01817
61,2
GD_SNP00206
GD_SNP01107
62,4
GD_SNP01200
GD_SNP00886
63,1
GD_SNP01277
63,2
GD_SNP01596*
64,6
10
GD_SNP01504
0,0
GD_SNP00253
3,5
4,5
CH04h02
5,2
GD_SNP01480
GD_SNP02281
9,9
GD_SNP01867
15,1
GD_SNP00351
11,7
GD_SNP00875
15,7
14,2
GD_SNP01543
GD_SNP01619
19,1
CONS67
24,0
26,0
GD_SNP01312
21,5
GD_SNP01713
26,7
GD_SNP00260
23,4
GD_SNP01912
30,3
CONS52
24,7
GD_SNP01112
32,0
GD_SNP01267
33,6
GD_SNP00185
33,9
GD_SNP00015
34,9
GD_SNP00690
34,1
GD_SNP00781
35,7
GD_SNP00735
36,0
GD_SNP01710
GD_SNP00176
GD_SNP00254
37,8
40,2
GD_SNP02252
GD_SNP00377
41,7
GD_SNP01832
GD_SNP02309
42,5
40,3
GD_SNP02179
GD_SNP00037
44,7
GD_SNP00034
GD_SNP02183
40,4
45,7
GD_SNP02465
GD_SNP00869
41,9
48,1
GD_SNP02072
48,6
55,7
GD_SNP00812
60,4
GD_SNP00307
GD_SNP00065
63,0
GD_SNP01570
63,4
GD_SNP00355
65,1
GD_SNP01961
71,8
74,6
GD_SNP00099
80,7
PPO_SNP
MdPPO_SSR10d
82,4
GD_SNP00258
83,3
HI08h12
83,4
GD_SNP01683
86,7
GD_SNP00405
94,9
Md-ACO1
11
GD_SNP01270
0,0
GD_SNP00116
1,8
GD_SNP01391*
4,8
GD_SNP01615
5,0
7,7
GD_SNP01795*
8,0
GD_SNP01907*
9,9
GD_SNP02212
10,9
GD_SNP02560
12,0
GD_SNP02537
14,3
GD_SNP02857
GD_SNP01052
18,3
GD_SNP02814
GD_SNP02089
18,4
GD_SNP00318
19,1
GD_SNP02326
20,8
GD_SNP02582
21,3
HI03b03
22,3
GD_SNP01798
23,1
GD_SNP01029
28,3
GD_SNP01995
30,9
GD_SNP01420
31,7
GD_SNP00747
32,6
GD_SNP01426
33,4
GD_SNP01022
39,6
GD_SNP00685
GD_SNP01056
48,2
40,7
GD_SNP00391
GD_SNP02800
53,6
GD_SNP00620
50,9
GD_SNP00123
53,8
GD_SNP01051
51,7
GD_SNP00113
53,9
GD_SNP01180
52,3
GD_SNP01739
55,1
GD_SNP00974
52,6
GD_SNP02475
56,0
GD_SNP00334
52,7
GD_SNP00212
GD_SNP01874*
56,7
57,9
GD_SNP02351
GD_SNP00338
64,0
GD_SNP00004
62,9
GD_SNP01855
68,5
GD_SNP02524
63,2
GD_SNP00915
69,8
HI06b06
63,7
GD_SNP00296
71,2
GD_SNP00773
79,6
GD_SNP01926
80,1
GD_SNP01465
82,1
GD_SNP00029
83,2
GD_SNP01540
12
13
GD_SNP00184*
0,0
GD_SNP00060
1,1
0,0
GD_SNP00025
GD_SNP00110
2,7
1,4
GD_SNP02856
GD_SNP01297
5,0
2,4
GD_SNP01767
6,2
GD_SNP00195
2,5
GD_SNP00248
9,1
GD_SNP02336
4,5
GD_SNP01733
9,2
GD_SNP01533
GD_SNP02214
9,3
HI04g05
18,7
GD_SNP02543
9,5
GD_SNP00581
GD_SNP00170
19,8
10,2
GD_SNP00922
GD_SNP01315
21,3
10,8
GD_SNP02054
GD_SNP02638
23,3
12,5
GD_SNP00531
23,4
GD_SNP02135
GD_SNP00128
14,9
23,6
GD_SNP00259
GD_SNP01507
18,7
GD_SNP01013
GD_SNP00623
29,6
23,8
GD_SNP02523
GD_SNP02002
30,5
24,7
GD_SNP02366
GD_SNP01536
33,0
26,4
GD_SNP00062
GD_SNP00340
34,4
27,7
GD_SNP02673
34,8
GD_SNP02028
28,6
CH03g04
39,5
GD_SNP01844
GD_SNP01900*
29,0
40,1
GD_SNP02032
GD_SNP01473*
30,0
42,2
GD_SNP00902
GD_SNP00453
30,4
46,8
GD_SNP02063
48,2
GD_SNP02069
50,8
GD_SNP00755
52,6
GD_SNP02452
GD_SNP01786
53,9
GD_SNP00994
55,3
GD_SNP01316
56,3
GD_SNP00350
57,0
GD_SNP01317
58,6
GD_SNP02890
58,7
GD_SNP01225
58,8
GD_SNP00748
59,7
GD_SNP01775
59,8
GD_SNP00770
65,2
GD_SNP02152
60,9
GD_SNP01899
14
GD_SNP00349
0,0
GD_SNP01131
1,8
GD_SNP00717
2,8
GD_SNP00115
6,6
6,9
GD_SNP00842
9,5
GD_SNP01666
11,0
CH03b06
12,2
GD_SNP01814
12,7
GD_SNP00329
14,1
GD_SNP01469
15,2
GD_SNP01827
16,8
GD_SNP01551
GD_SNP00076
24,0
GD_SNP01820
24,5
GD_SNP00831
25,8
GD_SNP01206
30,2
GD_SNP00017
32,9
GD_SNP00397
34,2
GD_SNP00429
38,2
GD_SNP00273
45,2
GD_SNP01229
46,3
GD_SNP01412
50,8
GD_SNP02080
GD_SNP00211
31,1
51,6
GD_SNP00972
GD_SNP00048*
38,2
55,9
GD_SNP01622
GD_SNP01512
39,3
56,6
GD_SNP02313
40,6
GD_SNP01811
GD_SNP01850
62,3
40,9
GD_SNP00498
GD_SNP00032
62,7
42,2
GD_SNP00171
GD_SNP00962
65,2
48,3
CONS80
GD_SNP02823
65,9
54,2
GD_SNP00213
GD_SNP02084
68,0
57,0
GD_SNP02904
GD_SNP02628
68,1
61,1
GD_SNP02079
Md-ACS1
69,2
62,5
GD_SNP00023
GD_SNP00241
70,9
GD_SNP01639
63,5
GD_SNP02417
GD_SNP00052
66,1
74,8
GD_SNP01133
77,5
GD_SNP01971
81,0
GD_SNP02045
15
16
GD_SNP00292
GD_SNP00091
0,0
0,0
CH05c06
CH04c06
1,1
1,5
GD_SNP00993
GD_SNP00268
2,3
GD_SNP01943
GD_SNP01205
2,4
2,5
GD_SNP00196
GD_SNP02075
4,9
CONS47
4,3
GD_SNP00290
5,6
GD_SNP01116
7,0
GD_SNP01241
6,8
GD_SNP00200
12,9
GD_SNP01403
7,5
GD_SNP00069
19,5
GD_SNP00088
8,4
GD_SNP00832
22,7
GD_SNP00328
12,2
GD_SNP01186
24,6
GD_SNP01932
13,3
GD_SNP00286
26,2
GD_SNP00262
GD_SNP01179
GD_SNP01456
13,4
26,4
GD_SNP00030
GD_SNP00515
15,9
28,6
GD_SNP01173
GD_SNP02512
37,9
18,2
GD_SNP00707
GD_SNP00287
40,4
GD_SNP00071
GD_SNP02172
19,4
40,7
CH05a04
GD_SNP01595
27,0
41,2
28,2
GD_SNP01730
41,4
HI03c05
29,4
GD_SNP01715
41,5
GD_SNP01800
32,3
GD_SNP00356
41,6
GD_SNP00682
37,2
GD_SNP01785
42,2
GD_SNP01044
38,2
GD_SNP02783
42,5
GD_SNP01269
38,8
GD_SNP00626
42,8
GD_SNP00401
42,9
GD_SNP01042
44,7
GD_SNP01842
GD_SNP02572
49,4
GD_SNP01438
52,5
GD_SNP00235
53,4
GD_SNP00379
53,8
GD_SNP00058
54,4
GD_SNP02678
57,9
GD_SNP00245
59,1
GD_SNP01752
81,3
GD_SNP00079
GD_SNP00525
82,0
GD_SNP01963
82,5
GD_SNP02660
GD_SNP01774
82,6
GD_SNP00236
83,6
GD_SNP01778
85,3
17
61,2
GD_SNP00438
