# Supplementary material for: A Multidisciplinary Approach Providing New Insight into Fruit Flesh Browning Physiology in Apple (Malus x domestica Borkh.)
Source: PLoS One. 2013 Oct 18;8(10):e78004. doi: 10.1371/journal.pone.0078004 (PMC3799748; doi:10.1371/journal.pone.0078004)
Supplement: Figure S6 — Nucleotide and aminoacid sequence of Md-PPO. Section “a” of the figure shows the Md-PPO nucleotide sequence of ‘Golden Delicious’ (>PPO_GD; accession number: KF561111) and ‘Braeburn’ (>PPO_Br; accession number: KF561112). In the two sequences the two SNPs are highlighted in bold black text. Section “b” shows the alignment of the predicted aminoacids in ‘Golden Delicious’ (GD_prot) and Braeburn (Br_prot). The aminoacid substitution conferred by SNP170 is shown in bold black text. (DOC) [file pone.0078004.s006.doc]

a

>PPO_GD

CCCAGCCCAACTTTGAGGACATGGGGAATTTTTACTCCG

CTGGTCGGGACCCCATATTTTTTGCACACCATTCGAATGTCGATCGAATGTGGA**G**TATTT

GGAAAACTCTTGGAGGTAAGAGAACTGATCTTACTGACTCGGACTGGTTGGACTCCGGAT

TCTTGTTTTACAACGAGAACGCAGAGTTAGTCCGAGTCAAGGTCAGGGACTGCTTGGAGA

CCAAAAATCTTGGGTATGTATACCAAGATGTGGACATTCCTTGGCTCAGCTCCAAGCCAA

CACCGCGAAGGGCGAAAGTTGCATTGAGCAAAGTAGCGAAGAAGCTGGGAGTTGCACACG

CAGCTGTTGCGTCGTCCAGCAAGGTGGTGGCAGGCACTGAGTTCCCGATAAGTCTGGGGT

CGAAGATAAGCACGGTGGTGAAGAGACCGAAGCAGAAGAAGAG**G**AGCAAGAAGGCCAAGG

AGGATGAGGAGGAGATATTGGTGATTGAGGGAATCGAGTTTGACAGGGACGTGGCTGTGA

GTTTTGATGAG

>PPO_Br

CCCAGCCCAACTTTGAGGACATGGGGAATTTTTACTCCG

CTGGTCGGGACCCCATATTTTTTGCACACCATTCGAATGTCGATCGAATGTGGA**C**TATTT

GGAAAACTCTTGGAGGTAAGAGAACTGATCTTACTGACTCGGACTGGTTGGACTCCGGAT

TCTTGTTTTACAACGAGAACGCAGAGTTAGTCCGAGTCAAGGTCAGGGACTGCTTGGAGA

CCAAAAATCTTGGGTATGTATACCAAGATGTGGACATTCCTTGGCTCAGCTCCAAGCCAA

CACCGCGAAGGGCGAAAGTTGCATTGAGCAAAGTAGCGAAGAAGCTGGGAGTTGCACACG

CAGCTGTTGCGTCGTCCAGCAAGGTGGTGGCAGGCACTGAGTTCCCGATAAGTCTGGGGT

CGAAGATAAGCACGGTGGTGAAGAGACCGAAGCAGAAGAAGAG**A**AGCAAGAAGGCCAAGG

AGGATGAGGAGGAGATATTGGTGATTGAGGGAATCGAGTTTGACAGGGACGTGGCTGTGA

GTTTTGATGAG

b

PPO_GD VRELILLTRTGWTPDSCFTTRTQSSESRSGTAWRPKILGMYTKMWTFLGSAPSQHREGRK 60

PPO_Br VRELILLTRTGWTPDSCFTTRTQSSESRSGTAWRPKILGMYTKMWTFLGSAPSQHREGRK 60

************************************************************

PPO_GD LHAKRRSWELHTQLLRRPARWWQALSSRVWGRRARWRDRSRRR**G**ARRPRRMRRRYWLRES 120

PPO_Br LHAKRRSWELHTQLLRRPARWWQALSSRVWGRRARWRDRSRRR**E**ARRPRRMRRRYWLRES 120

******************************************* ****************

PPO_GD SLTGTWLVLMX 131

PPO_Br SLTGTWLVLMX 131

***********
