# Supplementary material for: A Multidisciplinary Approach Providing New Insight into Fruit Flesh Browning Physiology in Apple (Malus x domestica Borkh.)
Source: PLoS One. 2013 Oct 18;8(10):e78004. doi: 10.1371/journal.pone.0078004 (PMC3799748; doi:10.1371/journal.pone.0078004)
Supplement: Table S1 — List of microsatellite marker primers designed on the contigs, related to the ten targeted PPO genes. The name, primer sequences, number of the contig and chromosome are given for each marker. (DOC) [file pone.0078004.s007.doc]

| ***NAME*** | ***primer_for*** | ***primer_rev*** | ***contig*** | ***CH*** |
| --- | --- | --- | --- | --- |
| PPO_SSR_ch2 | GTTTCGTTCCCCTCAACCTT | CCATTCCAACCAACAATTCC | MDC024616.45 | 2 |
| PPO_SSR_ch5a | GAGGGACCGCACACTAAAAA | TCCCACCCAATTATTCAAGC | MDC021736.76 | 5 |
| PPO_SSR_ch5b | CAACCCTTTTGGTTGTGAGG | GGGTCAACGGTCAAACTAGG | MDC015910.513 | 5 |
| PPO_SSR_ch5c | GCATGCCTATGAATTTTGTGAA | GCATCCTATTGTCATCCATCG | MDC022084.259 | 5 |
| PPO_SSR_ch5d | TGGACAAAAACCCGAAATGT | GGCTGTTAGTGCGTGTGTGT | MDC005212.234 | 5 |
| PPO_SSR_ch5e | TCCCACCCAATTATTCAAGC | GAGGGACCGCACACTAAAAA | MDC021736.141 | 5 |
| PPO_SSR_ch10a | TCCAAAAGAGCACACACCTG | ACTCCACCGTAGAGGCTTCC | MDC012681.207 | 10 |
| PPO_SSR_ch10b | CGCATACCGTAGGATCGTTT | CCTCAGGTGAATGGGTCTTT | MDC022200.129 | 10 |
| PPO_SSR_ch10c | CAAATTGATATATTTGAGAATGGGTA | AATTTAGGGTTTAATACTCACACCTC | MDC021722.194 | 10 |
| PPO_SSR_ch10d | TGCGTGTATCCTTCAATCCT | GCACGTGGCATGTTAGAGAT | MDC003244.401 | 10 |
